# Downregulation of PLIN2 in human dermal fibroblasts impairs mitochondrial function in an age‐dependent fashion and induces cell senescence via GDF15

**DOI:** 10.1111/acel.14111

**Published:** 2024-04-22

**Authors:** Antonio Chiariello, Luca Rossetti, Sabrina Valente, Gianandrea Pasquinelli, Manuela Sollazzo, Luisa Iommarini, Anna Maria Porcelli, Monica Tognocchi, Giuseppe Conte, Aurelia Santoro, Katarzyna M. Kwiatkowska, Paolo Garagnani, Stefano Salvioli, Maria Conte

**Affiliations:** ^1^ Department of Medical and Surgical Sciences (DIMEC) University of Bologna Bologna Italy; ^2^ Interdepartmental Centre “Alma Mater Research Institute on Global Challenges and Climate Change (Alma Climate)” University of Bologna Bologna Italy; ^3^ IRCCS Azienda Ospedaliero‐Universitaria di Bologna Bologna Italy; ^4^ Department of Pharmacy and Biotechnology (FABIT) University of Bologna Bologna Italy; ^5^ Department of Agriculture, Food and Environment University of Pisa Pisa Italy

**Keywords:** cell senescence, GDF15, human aging, lipid metabolism, mitochondrial dysfunction, PLIN2

## Abstract

Perilipin 2 (PLIN2) is a lipid droplet (LD)‐coating protein playing important roles in lipid homeostasis and suppression of lipotoxicity in different tissues and cell types. Recently, a role for PLIN2 in supporting mitochondrial function has emerged. PLIN2 dysregulation is involved in many metabolic disorders and age‐related diseases. However, the exact consequences of PLIN2 dysregulation are not yet completely understood. In this study, we knocked down (KD) PLIN2 in primary human dermal fibroblasts (hDFs) from young (mean age 29 years) and old (mean age 71 years) healthy donors. We have found that PLIN2 KD caused a decline of mitochondrial function only in hDFs from young donors, while mitochondria of hDFs from old donors (that are already partially impaired) did not significantly worsen upon PLIN2 KD. This mitochondrial impairment is associated with the increased expression of the stress‐related mitokine growth differentiation factor 15 (GDF15) and the induction of cell senescence. Interestingly, the simultaneous KD of PLIN2 and GDF15 abrogated the induction of cell senescence, suggesting that the increase in GDF15 is the mediator of this phenomenon. Moreover, GDF15 KD caused a profound alteration of gene expression, as observed by RNA‐Seq analysis. After a more stringent analysis, this alteration remained statistically significant only in hDFs from young subjects, further supporting the idea that cells from old and young donors react differently when undergoing manipulation of either PLIN2 or GDF15 genes, with the latter being likely a downstream mediator of the former.

## Graphical abstract

Perilipin 2 (PLIN2) plays a role in supporting a normal mitochondrial functionality. We showed that PLIN2 knockdown (KD) in human dermal fibroblasts caused a decline of mitochondrial function, an increased expression of the stress‐related mitokine GDF15 and the induction of cell senescence. Interestingly, the GDF15 KD abrogated the induction of PLIN2 KD‐associated cell senescence, suggesting that GDF15 could be the mediator of this phenomenon.

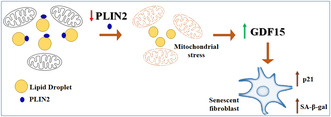

**Abbreviations**
BMPs: bone morphogenetic proteins
BSA: bovine serum albumin
CV: clear vacuoles
DV: dark vacuoles
ELISA: Enzyme‐linked immunosorbent assay
ER: endoplasmic reticulum
GDF11: growth differentiation factor 11
GDF15: growth differentiation factor 15
hDFs: human dermal fibroblasts
ISR: integrated stress response
KD: knock down
KO: knockout
LD: lipid droplet
NGS: next generation sequencing
OA: oleic acid
OCR: oxygen consumption rate
ohDFs: old human dermal fibroblasts
OXPHOS: oxidative phosphorylation
PI: propidium iodide
PLINs: Perlipins
RNAi: RNA interference
RT‐PCR: reverse transcriptase‐polymerase chain reaction
SASP: senescence‐associated secretory phenotype
SA‐β Gal: senescence associated‐beta galactosidase
siRNA: small interfering RNA
TBARS: thiobarbituric acid reactive substances
TEM: transmission electron microscopy
TGF‐β: transforming growth factor beta
UPRmt: mitochondrial unfolded protein response
yhDFs: young human dermal fibroblasts

## 1 INTRODUCTION

Lipid metabolism plays a pivotal role in homeostasis and survival of cells. In a great variety of mammalian cells, lipids are stored in intracellular structures known as lipid droplets (LDs), dynamic organelles involved in different important cellular processes. LDs are composed by a core of neutral lipids, such as cholesterol esters and triglycerides, surrounded by a monolayer of phospholipids and a large variety of proteins (Olzmann & Carvalho, 2019). Among these, the most abundant and best characterized are perilipins (PLINs), involved in lipid storage and LDs homeostasis. PLINs belong to a family of five proteins (from PLIN1 to PLIN5), each showing a tissue‐specific expression pattern in human body (Conte et al., 2016). Among PLINs, PLIN2 is thought to be fundamental in LDs biogenesis and lipolysis regulation and it is considered a key player of lipid metabolism in different tissues and cell types (Chang et al., 2006; Xu et al., 2019). PLIN2 dysregulation is involved in many diseases. As an example, we have found that high levels of PLIN2 are positively associated with muscle atrophy and sarcopenia (Conte et al., 2013), and that in vivo muscle‐specific PLIN2 knockdown (KD) in mouse induces an increase in skeletal muscle cross‐sectional area (Conte, Armani, et al., 2019). Other studies have indicated that PLIN2 expression is correlated with metabolic disorders, Type 2 diabetes, and hepatic steatosis (Ji et al., 2019; Zhang et al., 2018). Different cancers express altered levels of PLIN2 too, with a positive or negative association with overall survival depending on the type of cancer (Hayakawa et al., 2023; Zhang et al., 2021). Finally, PLIN2 was also found to be highly expressed in neurons of the gray matter from frontal and temporal cortex, hippocampus and cerebellum of old subjects, and patients with Alzheimer's disease or early tauopathy (Conte, Medici, et al., 2022).

It has been shown that PLIN2 knockout (KO) in C2C12 cells causes a LDs enlargement and increased lipolysis. Conversely, LDs disruption led to a reduced stability of PLIN2 protein, indicating that LDs and PLIN2 are tightly linked in a reciprocal way (Xu et al., 2019). Moreover, it is known that PLIN2 is rapidly degraded by the ubiquitin–proteasome system when it is detached from LDs (Xu et al., 2005).

Recently, a role for PLIN2 in regulating and sustaining mitochondrial function has emerged. In particular, LDs can interact with mitochondria, and PLIN2 (as well as PLIN1 and PLIN5) might be involved in this interaction (Cui et al., 2019; Yu et al., 2015). These LDs–mitochondria contacts are important for lipid utilization in mitochondrial β‐oxidation and are particularly abundant in tissues with a high metabolic rate (Cui & Liu, 2020). Another group showed that downregulation of PLIN2 in pancreatic β cells obtained from mice on high‐fat diet affects insulin secretion, decreases OXPHOS subunit expression, and reduces mitochondrial respiration (Mishra et al., 2021). Moreover, it has been recently shown that under stress conditions, mitochondrial p53 can interact with PLIN2 and this interaction can mediate an increase in LDs–mitochondria contacts, leading to lipid accumulation (Che et al., 2023). Mitochondrial dysfunction and cell senescence are intimately linked, and the former has been proven to cause the latter in a series of experimental models (Guan et al., 2019; Miwa et al., 2022; Wiley et al., 2016). Here we aim to understand whether PLIN2 downregulation induced cell senescence. We transiently downregulated PLIN2 in primary human dermal fibroblasts (hDFs) from both young (mean age 29 years) and old (mean age 71 years) subjects. In particular, in hDFs from young donors, PLIN2 KD resulted in decreased mitochondrial respiration, upregulated growth differentiation factor 15 (GDF15) expression and induction of cell senescence. GDF15 is a member of the TGF‐β superfamily that includes activins and BMPs and is considered a stress responsive cytokine with beneficial effects on health and lifespan in many model organisms. Moreover, GDF15 is a well‐known biomarker of primary mitochondrial dysfunction in muscle (Conte, Giuliani, et al., 2022; Wang et al., 2021). Interestingly, we have found that the simultaneous KD of both PLIN2 and GDF15 abrogated cell senescence induction. As a whole, our data suggest that PLIN2 KD‐induced cell senescence is likely mediated by GDF15, that emerges as a key player of stress response, as its depletion causes a global reshape of gene expression. Interestingly, these effects are particularly evident in cells from young subjects, suggesting that cells from old subjects are less responsive to mitochondrial stress, possibly due to chronically elevated GDF15 levels.

## 2 MATERIALS AND METHODS

### 2.1 Primary human dermal fibroblasts culture and in vitro KD

Human dermal fibroblasts (hDFs) were obtained from biopsies of sun‐protected areas (forearm) of different healthy subjects, divided in young adults (6 subjects, 2 women and 4 men: age range 25–34 years; mean 28.5 ± 3.6) and old people (5 subjects, 3 women and 2 men: age range 63–78 years; mean 70.6 ± 6.3). Cells were cryopreserved in liquid nitrogen using a medium composed by 10% dimethyl sulfoxide (DMSO) in fetal calf serum (FCS) and were available at Salvioli's Lab biobank. The study protocols were approved by Sant'Orsola‐Malpighi University Hospital Ethical Committee (EC), Bologna, Italy (EC 22/2007/U/Tes, approved 27/02/2007, amendment 14/06/2012).

hDFs were cultured in DMEM‐high glucose supplemented with 10% heat‐inactivated fetal calf serum (FCS), penicillin (100 units/mL), streptomycin (100 mg/mL), and 2 mM L‐glutamine (all from Sigma), in an incubator at 5% CO_2_, with a humidified atmosphere of 37°C. Cells between passages from sixth to tenth were used for the experiments.

PLIN2, PLIN3, and GDF15 KD were obtained by using the RNA interference (RNAi) technique. Predesigned siRNAs were purchased from Cohesion Biosciences. Three different siRNA for each transcript were provided and the optimal combination of siRNAs to obtain the best efficiency of downregulation was chosen after performing tests in a preliminary silencing experiment. For PLIN2 the chosen combination of siRNA oligos reached a silencing efficacy of about 70%–80%, for PLIN3 80%–90%, and for GDF15 60%–70%. Transfection was performed in 6‐well plates (125,000 cells/well) using ScreenFect siRNA reagent (ScreenFect GmbH) as reported in Chiariello et al. (2023) (Chiariello et al., 2023). After 24 h of incubation with the siRNA, the medium was replaced with fresh complete medium, and cells were harvested after further 72 h for subsequent RNA or protein extraction.

### 2.2 RNA extraction and real‐time RT‐PCR analysis

Total RNA was extracted from hDFs pellets with the EasyPure RNA kit (TransGen Biotech Co., Ltd). RNA quantification was performed using NanoDrop One Spectrophotometer (Thermo Scientific). For cDNA synthesis HIScript III RT SuperMix for qPCR (+gDNA wiper) kit was used (Vazyme Biotech), following manufacturer's instructions.

Gene expression analyses were performed through real‐time RT‐PCR using iTaq Universal Sybr Green Supermix (Bio‐Rad) ran on Rotor Gene Q 6000 System (Qiagen). A comparative analysis was performed. After testing different housekeeping genes, glyceraldehyde‐3‐phosphate dehydrogenase (GAPDH) and β‐actin were chosen as reference genes, due to their stable results over all samples. The relative expression ratio was obtained using the 2^−ΔΔCT^ method. All predesigned primers were obtained from Bio‐Rad (more information is available on www.bio‐rad.com/PrimePCR).

### 2.3 Protein extraction and western blotting analysis

Protein extraction from hDFs pellets was performed using RIPA buffer (Tris HCl pH 8 50 mM, NaCl 150 mM, Sodium deoxycholate 0.5%, SDS 0.1% and Triton X‐100 1%). Lysis was performed using the above described buffer with the addition of protease and phosphatase inhibitors (Sigma). Pellets were resuspended in the buffer, left in ice for 12 min vortexing a few times. Lysates were then centrifuged at 15,000 rpm for 15 min at 4°C and the supernatant was collected. Quantification was performed by Bradford's method in duplicate and isolated proteins were stored at −80°C until used.

Protein expression was analyzed by western blotting. Proteins (5–20 μg, depending on the primary antibody used) were separated on a 12% polyacrylamide gel. They were transferred to a nitrocellulose or PVDF membrane (Trans‐Blot Transfer, and Bio‐Rad) and then immunoblotted with primary antibodies: GAPDH, PLIN1, PLIN2, PLIN3, PLIN4, PLIN5, and VDAC1 (Table S1). GAPDH was used as loading control. Chemidoc XRS+ system (Bio‐Rad) was used for images acquisition. Densitometry analysis of bands intensity was performed using Fiji software.

### 2.4 Enzyme‐linked immunosorbent assay (ELISA)

GDF15 concentration was determined by ELISA assay performed on hDFs cell culture media by using Human GDF‐15 Quantikine ELISA Kit (R&D Systems, DGD150; intra‐assay and inter‐assay coefficient of variation were 0.4%–3.6% and 6%–8.1%, respectively; assay sensitivity was 4.39 pg/mL). 96‐well plates were analyzed by absorbance reading using SynergyTM fluorometer (Bio‐Tek Instruments, Winooski, Vermont, USA). Standard curve was constructed from a serial dilution of standard sample, according to manufacturer's instructions. Duplicate aliquots of each sample were analyzed for GDF15 concentration in two different plates.

### 2.5 Oleic acid treatment on cultured hDFs

125,000 cells/well were seeded in 6‐well plates. Cultured hDFs were treated with a final concentration of 200 μM oleic acid (OA)–0.6% bovine serum albumin (BSA) (Sigma‐Aldrich, O3008) for 24 h before cell collection and analyses. 24 h 0.6% BSA treatment was used as negative control.

### 2.6 Flow cytometric analyses

Flow cytometry was used to evaluate the amount of intracellular lipids, the protein levels of PLIN2 and PLIN3, cell cycle, and cell death. The analysis was performed using a four‐laser FACSymphony A1 (BD Biosciences, San Jose, CA) or three‐laser MACSQuant Analyzer MQ16 (Miltenyi Biotec, Bergisch Gladbach, Germany). Flow cytometry data were analyzed with DiVa nine (BD Biosciences) and MACSQuantify software (Miltenyi Biotec). In total, 10,000 events were recorded for each sample. Acquisition and analysis gates were set on fibroblasts based on forward (FSC) and side scatter (SSC) properties of cells. FSC and SSC were set in a linear scale. Unstained controls were included for each sample.

The content of intracellular lipids was evaluated by using the dye 4,4‐difluoro‐1,3,5,7,8‐pentamethyl‐4‐bora‐3a,4a‐diaza‐s‐indacene (BODIPY 493/503; Life Technologies). Cells were grown in 6‐well plates and incubated with 2 μM BODIPY for 20 min at 37°C. Cells were then detached, collected, resuspended in PBS, and analyzed.

Immunofluorescence was performed on fixed hDFs using primary antibodies against PLIN2 and PLIN3 (LifeSpan BioSciences, Inc). Briefly, pelleted cells were fixed in a solution consisting of IC Fixation Buffer (eBioscience^TM^, Thermo Fisher Scientific #00‐8222‐49) diluted 1:1 with PBS for 15 min at room temperature and permeabilized in 4% paraformaldehyde (PFA) in PBS for 15 min at room temperature. Then, cells were sequentially incubated for 15 min at 4°C with primary antibody 1:50 in Flow Cytometry Staining Buffer (eBioscienceTM, Thermo Fisher Scientific #00‐4222‐57) and secondary antibody (Alexa FluorTM 488 donkey anti‐rabbit antibody; Thermo Fisher Scientific #R37118) 1:20 in Flow Cytometry Staining Buffer. PBS washes and 5‐min centrifuges at 1600 rpm were performed between staining incubations.

For cell cycle analysis, propidium iodide (PI) (ApexBio Technology) staining was used. Briefly, cells were trypsinized and transferred into an Eppendorf tube, then fixed in 70% ethanol for 1 h at 4°C. Cells were pelleted with a 10 min centrifugation at 1500 rpm at 4°C. Pellet were washed with 1X PBS and then incubated overnight at 4°C with a solution of PI (200 μg/mL) and NP‐40 (0.15%) in PBS.

LIVE/DEAD Viability/Cytotoxicity Assay kit (Thermo Fisher Scientific) was used to determine viability of cells according to manufacturer's instructions. For both tests (cell cycle and viability) cells were assayed upon scramble or PLIN2 transfection.

### 2.7 Fluorescence microscopy analysis

Intracellular lipid content and mitochondrial network were also evaluated qualitatively by fluorescence microscopy. Cells were cultured on a coverslip. After reaching ~80% confluency, cells were stained with BODIPY 493/503 2 μM or MitoTracker Red 25 nM, respectively, for 20 min at 37°C. Cells were then washed and fixed in 4% paraformaldehyde for 15 min at room temperature. After fixation, the samples were mounted with Antifade Prolong reagent with DAPI (Life Technologies). The day after, cells were analyzed with Nikon Eclipse Ni fluorescence microscope.

### 2.8 Analysis of cell senescence

Cell senescence was evaluated histochemically with the Cell Senescence Detection Kit (Abcam) according to manufacturer's instructions. Briefly, cells cultured in 6‐well plates were fixed with the fixative solution provided by the kit and then stained with the staining solution mix that allowed to detect the β‐galactosidase activity, which is considered one of the senescence hallmarks. After an overnight incubation at 37°C, cells were observed using an inverted microscope.

Semiquantitative analysis of cell senescence was performed using Fiji software. At least five images were analyzed per each sample and the experiment was performed on three biological replicates for each age group. The percentage of β‐galactosidase‐positive cells out of the total number of cells was considered.

### 2.9 Lipid peroxidation evaluation by TBARS assay

The level of lipid peroxidation in cultured cells was evaluated by TBARS assay. Briefly, 200 μL of 30% trichloroacetic acid (TCA) were added to pelleted samples, followed by the addition of 1.5 mL of phosphate buffer (pH = 7) and 1.5 mL of 0.8% thiobarbituric acid (TBA). Samples were then incubated at 95°C for 20 min. After 10 min of cooling at 4°C, samples were centrifuged at 1500 rpm for 10 min at 4°C. After centrifugation, organic layer was taken, and absorbance analysis was performed with 530 nm excitation.

### 2.10 Transmission electron microscopy (TEM) analysis

For cell ultrastructural analysis, transmission electron microscopy was used. Cells were cultured in 6‐well plates and at the end point of the experiment were washed with PBS and fixed with 2.5% glutaraldehyde at room temperature for 20 min. Cells were then detached by scraper and transferred into microtubes before centrifugation. The pellets were then stored overnight at 4°C in the fixative solution. After that, cells were rinsed, post‐fixed in 1% buffered osmium tetroxide for 1 h at RT, and gradually dehydrated using ethanol at increasing concentrations. Lastly, samples were embedded in Araldite resin for sectioning with ultramicrotome. Ultrathin sections were counterstained with uranyl acetate and lead citrate. Processed samples were observed in a Philips CM100 transmission electron microscope (FEI Company, ThermoFisher, Waltham, MA, USA). Quantification of endoplasmic reticulum (ER), clear vacuoles (CV), and dark vacuoles (DV) was manually performed on digital ultrastructural images taken from each young hDFs cell experimental condition and analyzed using ImageJ analysis software. Ten images for each condition (Control, PLIN2 KD, GDF15 KD, and PLIN2 + GDF15 KD) were analyzed.

### 2.11 Mitochondrial oxygen consumption rate measurement

Mitochondrial oxygen consumption rate (OCR) was determined using the protocol described for Seahorse XFe Mito Stress Test Kit (Agilent #103015‐100). Briefly, 2750 cells/well were seeded in 100 μL of DMEM medium into XFe 96‐cell culture plates and incubated at 37°C and 5% CO_2_. Upon KD of PLIN2, complete growth medium was replaced with 180 μL of unbuffered XF medium (Agilent #103575‐100) supplemented with 10 mM glucose, 1 mM sodium pyruvate, 2 mM L‐glutamine, at pH 7.4. After three OCR baseline measurements, 1 μM oligomycin (O), 1.5 μM carbonyl cyanide‐p‐trifluoromethoxyphenylhydrazone (FCCP), 1 μM rotenone (R), and 1 μM antimycin A (A) were sequentially added to each well. FCCP concentration was optimized on hDFs by titration before the experiments. After each injection three measurements over time were performed, indicated as timepoints. Following the manufacturer's protocol (Divakaruni et al., 2014), basal OCR was calculated using the values at Timepoint 3 subtracted from the non‐mitochondrial OCR, namely values obtained at Timepoint 15, after antimycin A injection for which a complete block of the mitochondrial respiratory chain is achieved by Complex III inhibition. ATP‐linked OCR was calculated subtracting OCR values at Timepoint 3 from OCR values at Timepoint 4 after injection of the specific F_1_F_o_ ATP synthase inhibitor oligomycin. Respiration associated with proton leak was determined as OCR values after oligomycin injection (Timepoint 4) subtracted for the non‐mitochondrial OCR (Timepoint 15). Maximal OCR corresponded to the maximal value of respiration after FCCP injection subtracted for non‐mitochondrial OCR, while spare respiratory capacity was calculated as maximal OCR subtracted for basal respiration and represents the respiratory reserve. At the end of the assay, medium was removed and sulforhodamine B (SRB) assay was performed to determine protein content. Briefly, plates were incubated with 10% trichloroacetic acid (TCA) for 1 h at 4°C to fix the cells. Then, cells were washed five times with water. Completely dried plates were incubated with 0.4% SRB for 30 min at room temperature. Then, SRB was solubilized with 10 mM Tris and absorbance at 560 nm was determined using a Victor2 plate reader (Perkin‐Elmer). Each biological replicate experiment (*n* ≥ 4) included measurements from at least six wells. Data (pmol/min) were normalized to blank corrected SRB absorbance.

### 2.12 RNA library preparation and sequencing

Genome‐wide transcriptome libraries were constructed on RNA extracted from hDFs from young and old subjects transfected with GDF15 and scramble siRNA.

The libraries were prepared with 150 ng–1 μg of total RNA using Illumina stranded mRNA prep ligation kit (Illumina) and dual indexes. Briefly, the purified and fragmented mRNA was converted to cDNA and amplified using Illumina primers containing dual indexes for each sample. Each library was subjected to quality control and quantification on Agilent Bioanalyzer using Agilent High Sensitivity DNA kit. An equal amount of libraries was pooled together and the library pool was re‐quantified with Agilent High Sensitivity DNA kit and sequenced on Illumina NextSeq500 platform using NextSeq 500/550 High Output Kit v2.5 (Illumina).

### 2.13 Quality control of NGS data, read mapping, and differential gene expression analysis

Raw base‐call data generated from the Illumina NextSeq500 system were demultiplexed by Illumina BaseSpace Sequence Hub (https://basespace.illumina.com/home/index) and converted to FASTQ format. After a quality check, which was performed with FastQC tool (https://www.bioinformatics.babraham.ac.uk/projects/fastqc/), the adapter sequences were trimmed using Cutadapt. Reads were aligned to human reference genome hg38 using STAR tool. To reconstruct the RNA‐Seq transcripts and quantify the expression, StringTie (v.2.1.7) was used. Differential expression analysis was performed in R environment (v.4.1.2) using Bioconductor's DESeq2 package.

In order to identify differential expression, linear model testing that logFC differed from 0 was generated and obtained *p* values were adjusted for multiple testing with Benjamini–Hochberg (BH) algorithm. Genes with nominal *p* value <0.05 and absolute value or fold change after binary logarithm transformation (|logFC|) >1 were considered as significant.

### 2.14 Statistical analysis

SPSS Statistics 28.0 software was used for statistical analyses. First, all data were analyzed by Shapiro–Wilk test for the normality assumption. Accordingly, data were principally analyzed by using Student's *t* test to determine differences between treated cells (siRNA, OA) and control groups. Mann–Whitney *U* test was used for the ELISA assay. Data are expressed as mean ± SE. *p* values below 0.05 were considered statistically significant.

## 3 RESULTS

### 3.1 PLIN2 and PLIN3 are expressed in hDFs but only PLIN2 is affected by intracellular lipid accumulation

**FIGURE 1:**
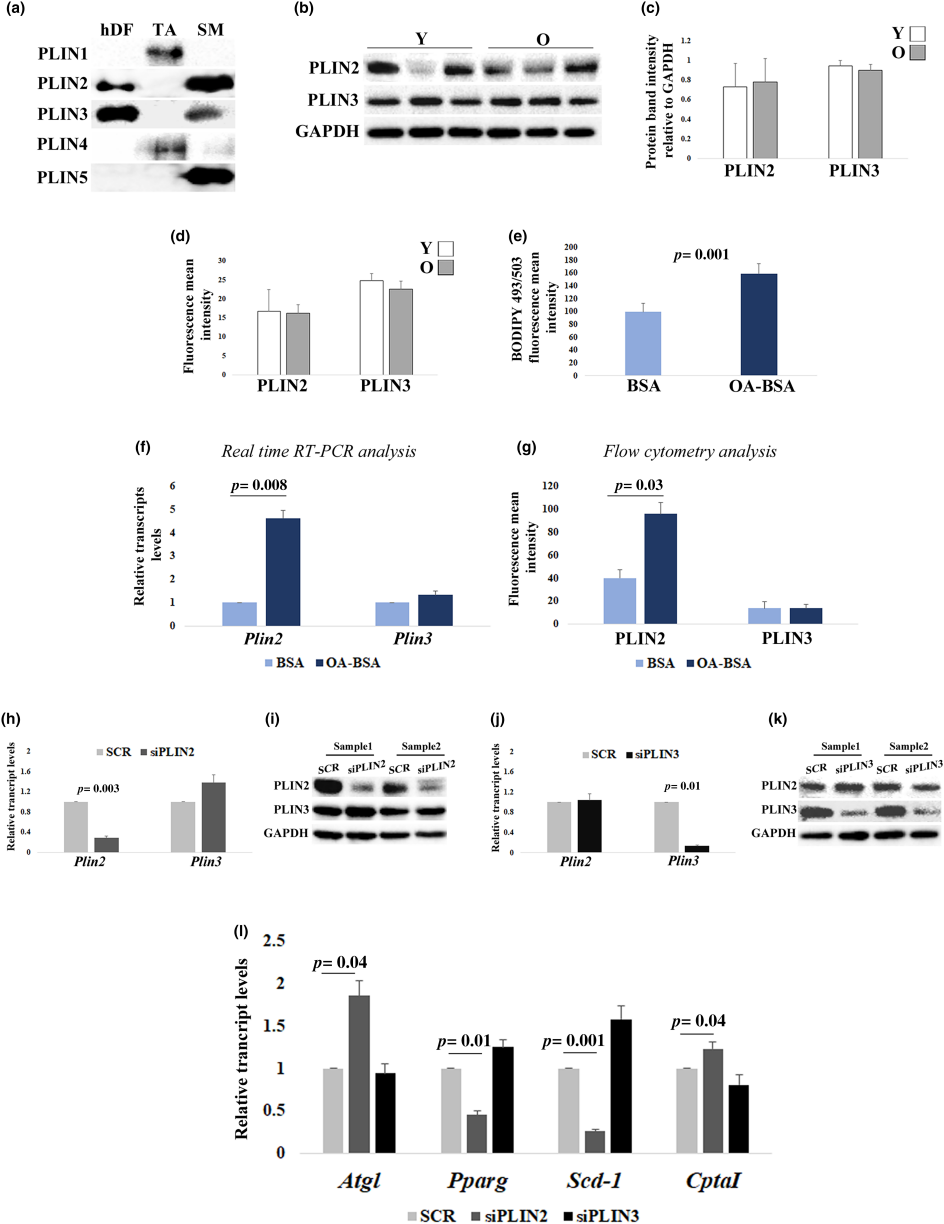
Perilipins (PLINs) expression analyses and effects of PLIN2 or PLIN3 downregulation in human dermal fibroblast (hDFs) from young (Y) and old donors (O). (a) Representative immunoblotting image of PLINs in a sample of hDFs. Adipose tissue (AT) and skeletal muscle (SM) were used as positive control for PLINs expression. (b and c) Western blotting analysis of PLIN2 and PLIN3 in hDFs from three young (Y) and three old (O) subjects. PLIN2 and PLIN3 levels are normalized to GAPDH levels. (d) Flow cytometry analysis of PLIN2 and PLIN3 levels in hDFs from three Y and three O. (e) Flow cytometry analysis of BODIPY 493/503 fluorescence intensity in three hDFs treated with oleic acid–BSA (OA‐BSA) compared to three controls (BSA). (f) Real‐time RT‐PCR analysis of *Plin2* and *Plin3* expression in 11 hDFs samples, from both Y and O, treated with BSA or OA‐BSA. (g) Flow cytometry analysis of PLIN2 and PLIN3 levels after OA‐BSA treatment in three hDFs samples. (h–k) Real‐time RT‐PCR and western blotting analysis of PLIN2 and PLIN3 after (h and i) PLIN2 downregulation (siPLIN2) and (j and k) PLIN3 downregulation (siPLIN3), compared to controls (SCR). (l) *Atgl, Pparg*, *Scd‐1*, and *CptaI* real‐time RT‐PCR analysis after siPLIN2 and siPLIN3 treatment in11 hDFs samples, from both Y and O. Data are expressed as mean ± SE. Student's *t* test was applied.

We have characterized the expression of PLINs in hDFs by western blotting and observed that only PLIN2 and PLIN3 are detectable in hDFs (Figure 1a). Since our previous studies demonstrated that PLIN2 (in both skeletal muscle and brain tissues) and PLIN3 (in brain tissue) are expressed at higher levels in old subjects with respect to young (Conte et al., 2013; Conte, Medici, et al., 2022), we then sought to investigate whether in hDFs also the expression of PLIN2 and PLIN3 was modulated by aging. We quantified the protein levels of PLIN2 and PLIN3 by western blotting analysis and flow cytometry. The analyses performed in three lines from young subjects (yhDFs) and three from old subjects (ohDFs) indicated that PLIN2 and PLIN3 expression in these cells do not change with age (Figure 1b–d). Notably, hDFs from young subjects seem to have a higher interindividual variability with respect to those from old subjects.

In order to investigate whether these PLINs can be induced by fatty acid accumulation, we analyzed the expression of PLIN2 and PLIN3 upon treatment of both yhDFs and ohDFs with 200 μM of oleic acid (OA) for 24 hours. BODIPY 493/503 staining was used to evaluate intracellular neutral lipid accumulation. After 24 hours of OA treatment, BODIPY 493/503 fluorescence intensity resulted significantly increased (Figure 1e), confirming that OA treatment promotes intracellular lipid accumulation in hDFs. Interestingly, upon OA treatment, only PLIN2 but not PLIN3 levels increased both at transcriptional and protein levels, thus suggesting that PLIN2 but not PLIN3 expression is responsive to fatty acid accumulation (Figure 1f,g).

### 3.2 PLIN2 but not PLIN3 KD leads to a reduction of LDs content and changes of the expression of lipid metabolism‐associated genes

To better understand the role of PLIN2 and PLIN3 in hDFs, we have performed a gene KD with a siRNA combination targeting PLIN2 and another one targeting PLIN3. Silencing efficacy was confirmed by both real‐time RT‐PCR and western blotting analyses (Figure 1h–k). The same analyses also indicated that PLIN2 KD did not influence the expression levels of PLIN3, and vice versa, suggesting that PLIN2 and PLIN3 do not compensate each other. Interestingly, only upon siPLIN2 transfection hDFs appeared to be smaller and possibly fewer in number with respect to scramble‐transfected hDFs, with a circle‐shaped morphology rather than the classical elongated spindle‐shaped (Figure S1a–c). In order to ascertain whether these phenotypic changes may be a reflection of altered cell survival or proliferation, we evaluated these parameters by flow cytometry. In particular, we performed cell cycle analysis by staining hDFs with propidium iodide (PI). No changes in G0/G1, S, or G2 phases were noticed, based on the abundance of the relative fluorescence peaks (Figure S1d–g). Furthermore, the same analysis can tell about the level of apoptosis, evaluated as events having a fluorescence intensity lower than the G0/G1 peak (Sub‐G0). No increase in the percentage of apoptotic events after PLIN2 downregulation was noticed. Moreover, a LIVE/DEAD Viability Assay was also performed. No relevant phenomena of cell death were observed, with no differences between scramble and PLIN2 KD samples (Figure S1h,i). These data indicate that the transfection protocol may have some phenotypic effects, however without affecting cell cycle and viability.

In order to clarify whether PLIN2 or PLIN3 downregulation induced changes in lipid metabolism, we performed a real‐time RT‐PCR analysis in yhDFs and ohDFs KD for either PLIN2 or PLIN3, compared to scramble‐transfected control, to evaluate the transcript levels of adipose triglyceride lipase (*Atgl*), peroxisome proliferators–activated receptor g (*Pparg*), stearoyl‐CoA desaturase‐1 (*Scd‐1*), and carnitine palmitoyltransferase 1A (*CptaI*), four genes involved in lipolysis, LD biogenesis and lipid accumulation, and fatty acid oxidation, respectively (Aljohani et al., 2019; Cerk et al., 2018; Janani & Ranjitha Kumari, 2015; Schlaepfer & Joshi, 2020). A significant increase in *Atgl* and *CptaI* transcript levels and a significant decrease of *Pparg* and *Scd‐1* transcripts were observed in PLIN2 KD hDFs compared to scrambles, while no changes were detected upon PLIN3 KD, thus suggesting that PLIN2 but not PLIN3 has a role in the regulation of lipid metabolism in hDFs (Figure 1l).

**FIGURE 2:**
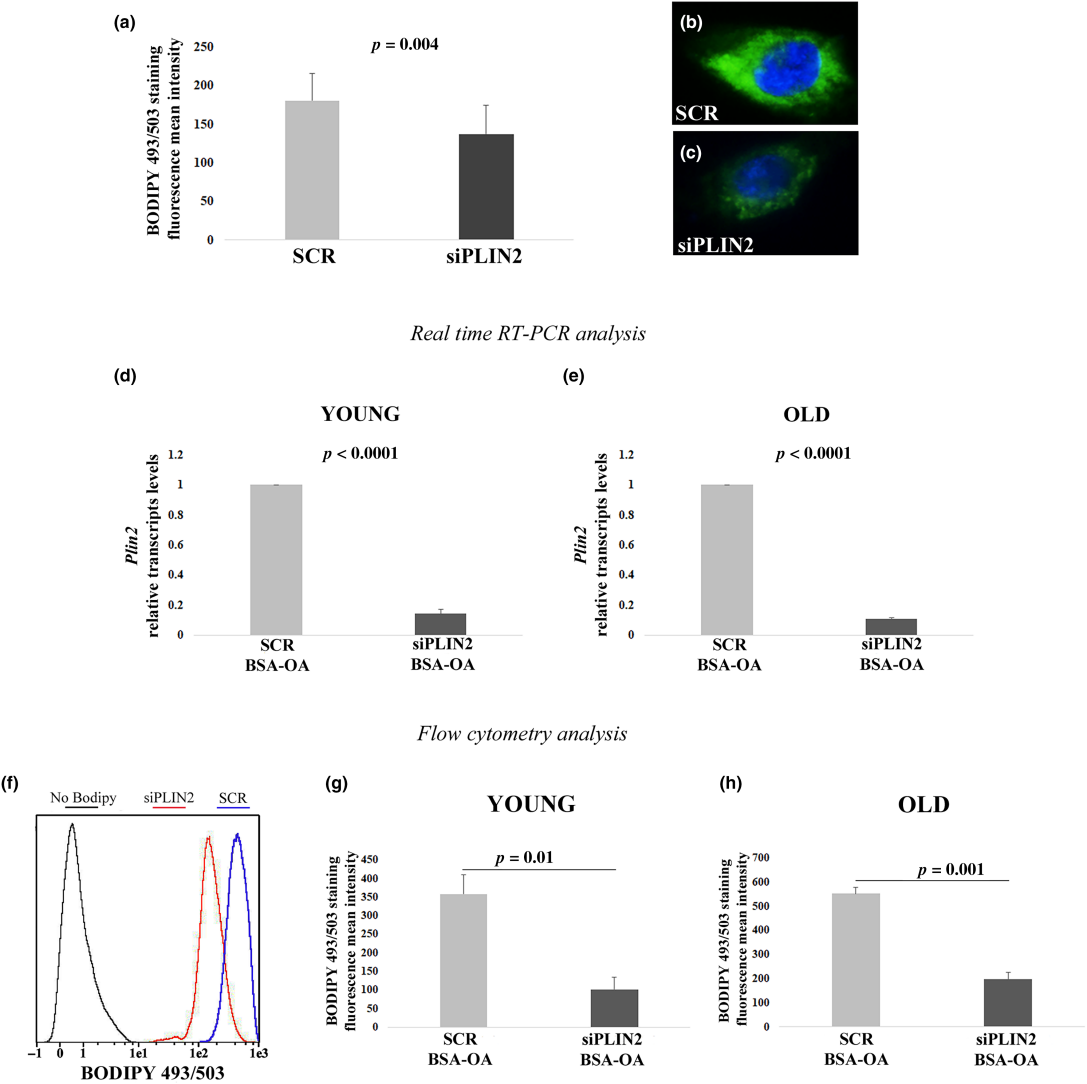
Flow cytometry analyses and real‐time RT‐PCR after siPLIN2 and OA‐BSA treatment. (a) BODIPY 493/503 fluorescence mean intensity quantification after siPLIN2 treatment in three hDFs samples from young donors (Y). (b and c) Representative fluorescence microscopy images of hDFs treated with siPLIN2 or scramble siRNA (SCR) and stained with BODIPY 493/503 (green). (d and e) Real‐time RT‐PCR analysis of PLIN2 expression after siPLIN2 and OA‐BSA treatment in hDFs from (d) six young and (e) five old donors. (f–h) Flow cytometry analysis of BODIPY 493/503 intensity after siPLIN2 and OA‐BSA treatment, in hDFs from (g) three young and (h) three old donors. Data are expressed as mean ± SE. Student's *t* test was applied.

In the light of these results, we then investigated whether the downregulation of PLIN2 also altered the levels of LDs. Therefore, we analyzed the content of LDs in PLIN2 KD hDFs with flow cytometry using BODIPY 493/503 staining. Flow cytometry analysis revealed that the mean fluorescence intensity of BODIPY 493/503 significantly decreases in PLIN2 KD cells compared to controls (scramble) (Figure 2a). This result was also confirmed by fluorescence microscopy, where the BODIPY 493/503 fluorescence appeared fainter and less diffused in the cytoplasm of PLIN2 KD cells compared to scramble‐treated cells (Figure 2b,c). The importance of PLIN2 for the accumulation of intracellular lipids was further confirmed by experiments of OA treatment in PLIN2 KD hDFs (Figure 2d–h). In fact, the intensity of BODIPY 493/503 fluorescence in OA‐treated hDFs significantly decreased upon PLIN2 KD in both yhDFs and ohDFs, suggesting that PLIN2 is not dispensable for LDs accumulation in hDFs (Figure 2f–h).

### 3.3 PLIN2 downregulation leads to mitochondrial stress

**FIGURE 3:**
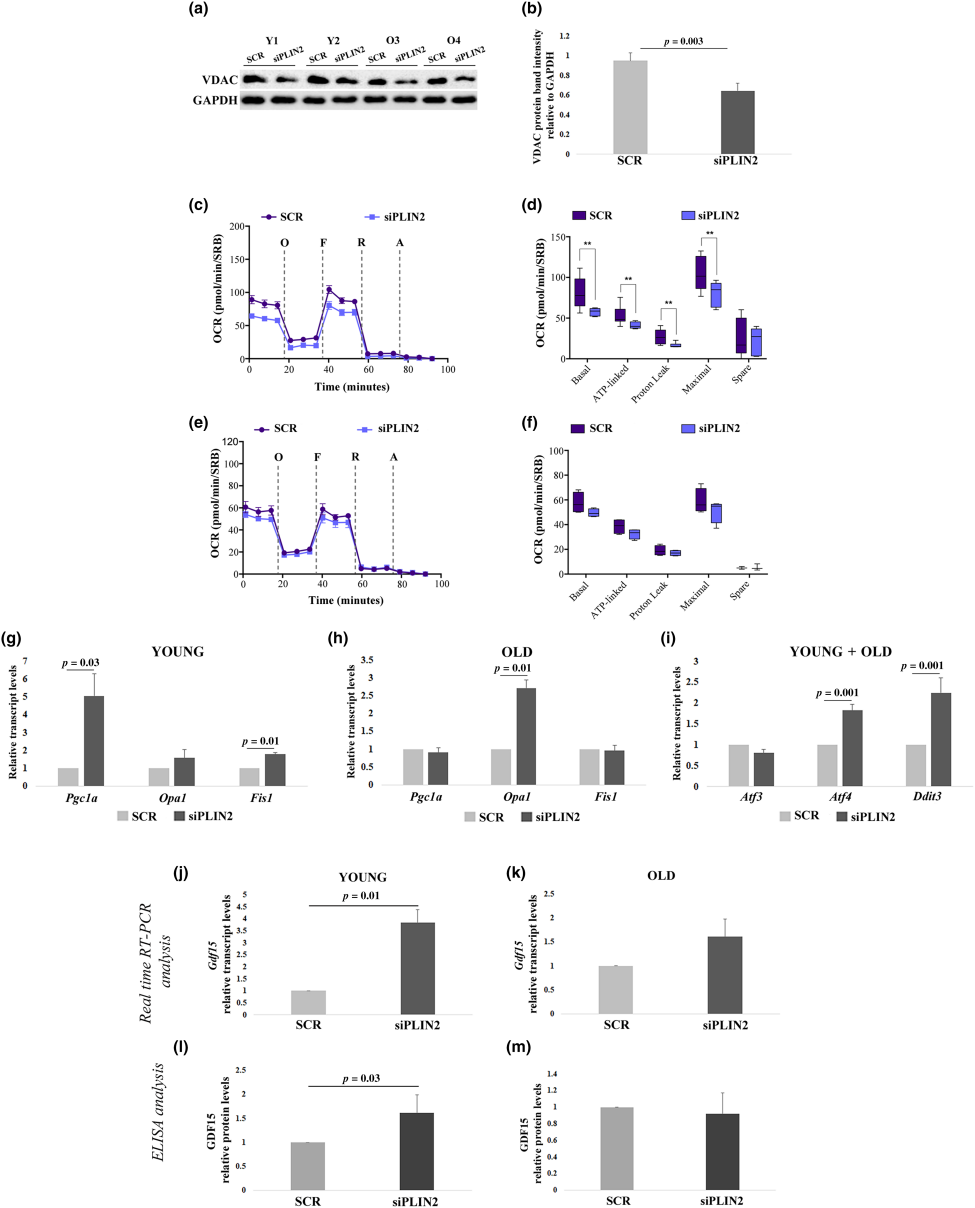
Mitochondrial functionality and expression analyses in hDFs form young (Y) and old (O) donors after PLIN2 downregulation. (a and b) Representative immunoblotting image and quantification of VDAC1 in four hDFs, two from Y and two from O donors, after siPLIN2 treatment compared to controls (SCR). (c–f) MitoStress Test in hDFs from three Y and three O donors after siPLIN2 treatment compared to controls using Seahorse XFe96 platform. (c,e) Oxygen consumption rate (OCR) profile of hDFs from Y (c; *n* ≥ 3) and O (e; *n* ≥ 3) determined upon injection of 1 μM oligomycin (O), 1.5 μM FCCP (F), 1 μM rotenone (R), and 1 μM antimycin A (A) in Seahorse XFe medium. FCCP concentration was previously determined by titration. Data are normalized on SRB absorbance. (d,f) Determination of different OXPHOS parameters: basal respiration, ATP‐linked respiration, proton leak, maximal OCR and spare respiratory capacity of hDFs from Y (d) and O (f) calculated from the data obtained in OCR profiles. (g and h) Real‐time RT‐PCR analyses of *Pgc1α*, *Opa1*, and *Fis1* expression in hDFs from (g) six Y and (h) five O donors. (i) Real‐time RT‐PCR analysis of *Atf3*, *Atf4*, and *Ddit3* expression after siPLIN2 treatment in 11 hDFs samples from both Y and O donors (young+old) grouped together, as the gene expression fold changes in PLIN2 KD samples compared to controls (scramble) were comparable between hDFs from young and elderly donors. (j,k) Real‐time RT‐PCR analysis of *Gdf15* expression after siPLIN2 treatment in hDFs from (j) six Y and (k) five O donors. (l,m) ELISA analysis of GDF15 secretion in the culture media of hDFs from (l) six Y and (m) five O donors, expressed as fold change compared to the scramble‐treated samples (SCR). Data are expressed as mean ± SE. Student's *t* test was applied. For ELISA analysis, Mann–Whitney *U* test was applied.

Considering that only PLIN2 seems to be important for lipid metabolism control, and based on previous results indicating that PLIN2 downregulation causes a mitochondrial respiration impairment (Mishra et al., 2021), we sought to investigate whether PLIN2 KD caused a mitochondrial defect also in hDFs. Indeed, mitochondrial mass estimated by western blotting of Voltage‐dependent Anion Channel 1 (VDAC1) resulted lower in PLIN2 KD yhDFs and ohDFs (Figure 3a,b). Moreover, PLIN2 KD induced an overall reduction of mitochondrial oxygen consumption rate (OCR), although to a different extent in the two donor groups (Figure 3c–f). In particular, basal, ATP‐linked, proton‐leak and maximal OCRs were significantly reduced upon PLIN2 KD in yhDFs (Figure 3c,d), indicating a general respiration impairment when PLIN2 is downregulated. A similar but not significant trend can be observed in ohDFs (Figure 3e,f), where basal, maximal and ATP‐linked OCRs were found slightly reduced upon PLIN2 KD, indicating that PLIN2 plays a regulatory role on the mitochondrial respiratory chain function mainly in yhDFs. To note, ohDFs showed a lower respiration when compared to yhDFs (Figure S2a), confirming an age‐related decline in mitochondrial functionality. To further investigate the involvement of mitochondria as target of PLIN2 KD, we explored the expression of representative genes of the mitochondrial dynamics. The expression of *Pgc1a*, *Opa1*, *Fis1*, involved in mitochondrial biogenesis, fusion and fission, respectively, was analyzed (Figure 3g,h). The expression levels of *Pgc1a*, *Opa1*, and *Fis1* were significantly higher in hDFs from young with respect to old donors at basal level (Figure S2b–d). However, upon siPLIN2 in yhDFs a significant increase in *Pgc1a* and *Fis1* expression was found; at variance in ohDFs a significant increase in *Opa1* expression was found. This suggests that cells from donors of different age likely implement different strategies to cope with the effects of PLIN2 downregulation, increased turnover or increased fusion, respectively.

The increased expression level of *Pgc1a* in yhDFs seems to be in contrast with the decrease in the expression level of *Pparg* (Figure 1l), as it is known that PPARg together with PGC1a is involved in mitochondrial biogenesis (Jamwal et al., 2021). However, it is possible that other PPAR members are involved in sustaining mitochondrial biogenesis in hDFs: as an example, PPARa is highly expressed in oxidative tissues and regulates genes involved in the regulation of energy homeostasis and it cooperates with PGC1a to regulate mitochondrial biogenesis (Christofides et al., 2021). Accordingly, *Ppara* expression level was significantly increased upon PLIN2 KD in yhDFs with respect to scramble control (Figure S3). Furthermore, staining with MitoTracker Red probe (which offers a qualitative glance of mitochondrial network) accordingly suggested that, upon PLIN2 KD, mitochondria from ohDFs appear more elongated with respect to those from yhDFs, suggesting that a phenomenon of mitochondrial fusion may possibly occur in the former (Figure S4). Further quantitative analyses would be needed to formally prove this hypothesis. Mitochondrial dysfunction is accompanied by a mitochondrial stress response. Accordingly, we observed a significantly increased expression, in both yhDFs and ohDFs, of transcription factors typically involved in the mitochondrial unfolded protein response (UPR^mt^), such as *Atf4* and *Ddit3*, even though *Atf3* expression, another transcription factor involved in the UPR^mt^, did not change (Figure 3i). These transcription factors are also able to promote the expression of a prototypical mitokine, GDF15, a member of transforming growth factor‐β (TGF‐β) superfamily whose expression increases in response to cellular stress and mitochondrial impairment (Conte, Giuliani, et al., 2022; Durieux et al., 2011). *Gdf15* transcript resulted upregulated in both yhDFs and ohDFs upon PLIN2 KD, even though statistical significance was reached only in yhDFs (Figure 3j,k). As expected, *Gdf15* expression levels were higher in ohDFs with respect to yhDFs (Figure S2e). To clarify whether PLIN2 KD‐induced *Gdf15* increase was specific, we also evaluated the expression of *Gdf11*, another member of the TGF‐β superfamily playing a crucial role in maintaining mitochondrial homeostasis in stress conditions (Zhao et al., 2020). Upon PLIN2 downregulation, the expression of *Gdf11* did not change in both yhDFs and ohDFs (Figure S5). This result suggests that the mitochondrial stress response following PLIN2 KD is specifically associated to *Gdf15*. In order to evaluate whether *Gdf15* gene expression positively correlated with the amount of secreted protein in the culture media, we then performed an ELISA assay on the supernatant of hDFs upon PLIN2 KD. To eliminate interindividual variability, the increment over the basal level of GDF15 concentration was calculated for each cell line. Accordingly, the increment of GDF15 concentration upon PLIN2 KD resulted statistically significant in yhDFs (Figure 3l,m). However, it is to note that the ohDFs featured higher basal GDF15 levels with respect to young ones in agreement with our previous results (Conte, Ostan, et al., 2019) (Figure S6).

We have also analyzed *Plin2* expression upon GDF15 KD in both yhDFs and ohDFs to clarify whether a feedback loop may exist between the two genes. Upon GDF15 KD, *Plin2* expression levels did not change, suggesting that there is no feedback effect of GDF15 on *Plin2* expression (Figure S7).

### 3.4 PLIN2 downregulation promotes cell senescence

**FIGURE 4:**
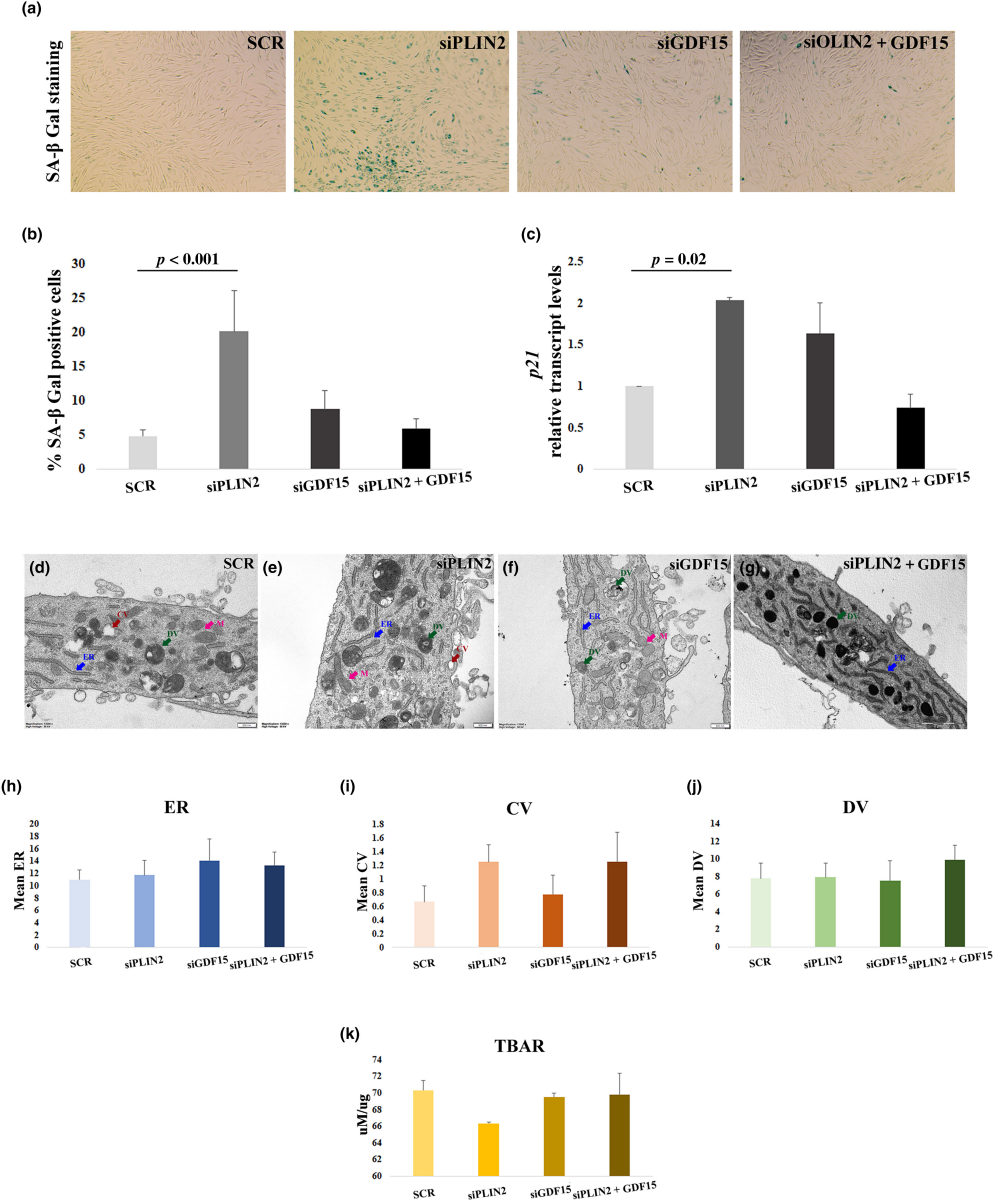
Cell senescence induction and transmission electron microscopy (TEM) analysis in hDFs from young donors. (a) Representative images of β‐galactosidase (SA‐β Gal) staining in hDFs after PLIN2 (siPLIN2), GDF15 (siGDF15), and PLIN2 and GDF15 combined (siPLIN2 + GDF15) and scramble (SCR) siRNA treatment. (b) Quantification with Fiji software of the percentage of senescent cells after experiment as in (a). At least five images per sample were used for quantification. The experiment was performed in three cell lines. (c) Real‐time RT‐PCR analysis of *p21* expression in six hDFs samples, after siPLIN2, siGDF15, siPLIN2 + siGDF15, or SCR treatment. (d–g) Representative images of TEM analysis after PLIN2 (siPLIN2), GDF15 (siGDF15), and PLIN2 and GDF15 combined (siPLIN2 + GDF15) and scramble (SCR) siRNA treatment. Arrows indicate endoplasmic reticulum (ER, blue), clear vacuoles (CV, red), dark vacuoles (DV, green), and mitochondria (M, magenta). (h–J) Quantification of (h) ER, (i) CV, and (j) DV. (k) Thiobarbituric acid reactive substances (TBARS) analysis. Data are expressed as mean ± SE. Student's *t* test was applied.

Considering that PLIN2 KD in yhDFs induces mitochondrial dysfunction, which in turn is linked to cell senescence, we wondered whether PLIN2 downregulation was able to induce cell senescence in these cells. We then performed β‐galactosidase (β‐Gal) assay upon PLIN2 KD. β‐Gal assay revealed that PLIN2 downregulation strongly induces cell senescence in yhDFs (Figure 4a,b) and this result was also confirmed by the increase in the expression level of *p21*, known for its role in the induction of cell cycle arrest and cell senescence (Kumari & Jat, 2021), analyzed by real‐time RT‐PCR (Figure 4c). As expected, ohDFs had a higher base‐line level of cell senescence (data not shown). As mitochondrial dysfunction leads to an increase in GDF15 expression, we wondered whether the increase in cell senescence upon PLIN2 KD was mediated by GDF15. We then performed GDF15 KD as well as a double KD of PLIN2 and GDF15 (PLIN2 + GDF15 KD) in yhDFs. Upon GDF15 KD, we observed a nonsignificant increase in cellular senescence and, strikingly, in PLIN2 + GDF15 KD yhDFs, the level of cell senescence was similar to that of scramble‐transfected yhDFs (Figure 4a–c), suggesting that the expression of GDF15 is fundamental to trigger PLIN2 KD‐induced cell senescence.

We then wondered whether GDF15 KD was able to induce changes in ultrastructural morphology or metabolism of hDFs that can account for the observed blockade of cell senescence. We thus performed a TEM analysis of hDFs from young donors (Figure 4d–j). We did not observe significant differences between PLIN2 KD, GDF15 KD and PLIN2 + GDF15 KD as compared with scramble‐transfected controls in terms of number of endoplasmic reticulum (ER) cisterns, as well as clear and dark vacuoles (CV and DV, respectively), suggesting that no big differences in terms of macroautophagy are present between treatments (Figure 4h–j). It is however to note that a remarkable increase in ER global content is visible in yhDFs upon GDF15 depletion, as suggested by Figure 4f. As oxidative stress is able to induce cell senescence too, we have also performed a test to evaluate the global burden of oxidative stress by measuring Thiobarbituric Acid Reactive Substances (TBARS) (Martín‐Fernández et al., 2022). No significant differences were noticed upon PLIN2 KD, GDF15 KD, or PLIN2 + GDF15 KD as compared to scramble in yhDFs (Figure 4k). As a whole, it was not possible to obtain evidence of gross modifications of cellular ultrastructure or oxidative stress to account for the GDF15 KD‐mediated blockade of cell senescence.

### 3.5 GDF15 KD determines a global change in gene expression

**FIGURE 5:**
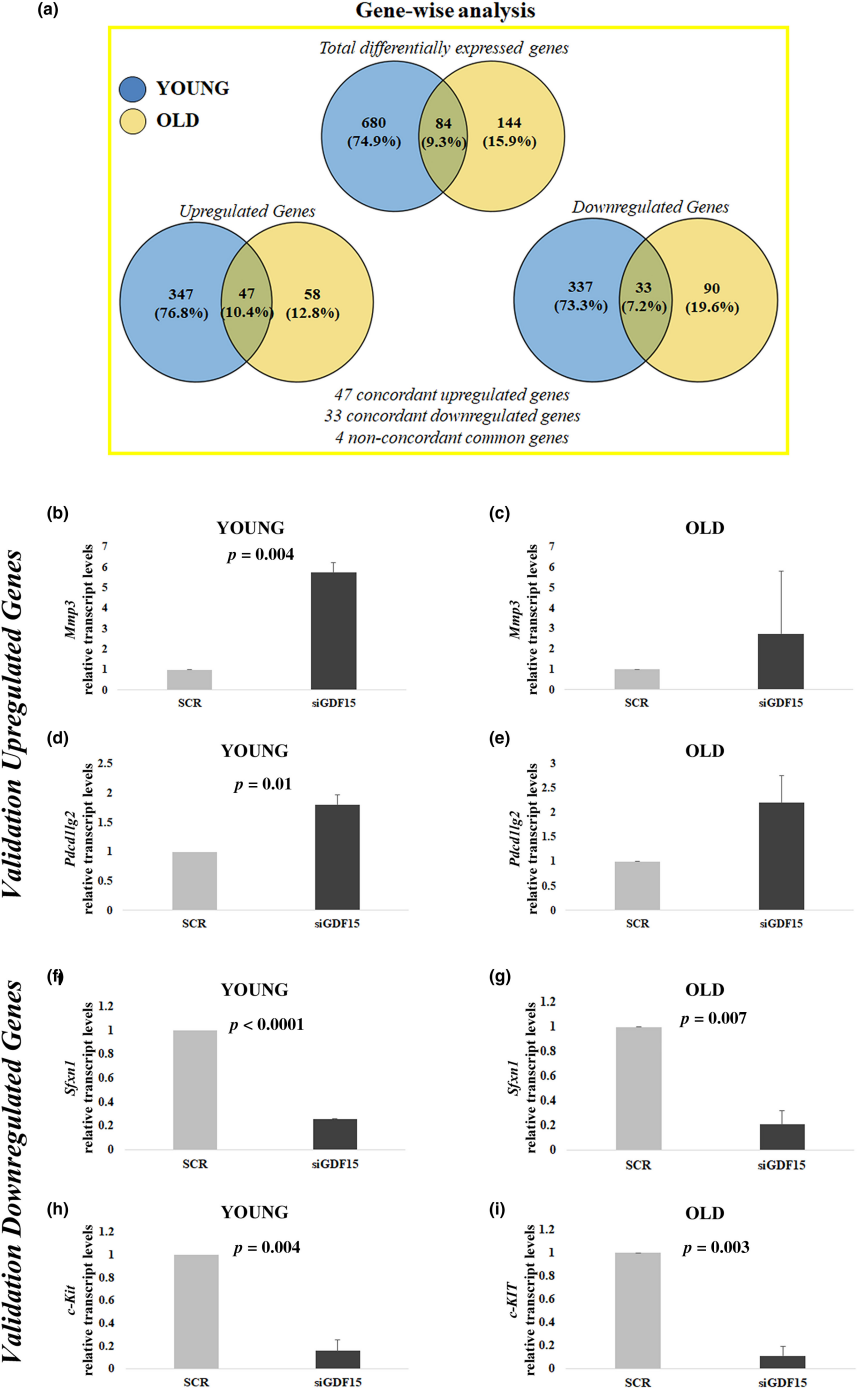
Whole‐genome RNA‐Seq analysis. (a) Representation of total and common differentially expressed genes in hDFs from three young and three old donors after GDF15 knockdown. (b–i) Validation of selected upregulated and downregulated genes (*Mmp3, Pdcd1lg2, Sfxn1*, and *c‐Kit*) emerged from RNA‐Seq, by real‐time RT‐PCR. Data are expressed as mean ± SE. Student's *t* test was applied.

It has been reported that GDF15 KD itself is able to induce cell senescence (Wedel et al., 2023). It is however to note that such effect was evaluated only by changes of the expression of SASP components, that were more pronounced in late passages DFs (Wedel et al., 2023). We thus wondered whether GDF15 depletion was per se able to modify gene expression patterns in a possibly age‐dependent fashion in hDFs. To answer this question, we then performed a whole genome RNA‐Seq in yhDFs and ohDFs upon GDF15 KD. As a whole, the expression of a total of 912 genes resulted modified upon GDF15 KD with a nominal *p* < 0.05: 452 resulted upregulated, while 460 resulted downregulated. Interestingly, 84 are shared between yhDFs and ohDFs, 80 of which change concordantly (Figure 5a). The large majority of gene expression changes is thus private and regards either yhDFs or ohDFs, indicating that cells from subjects of different age react differently to the depletion of GDF15. Interestingly, only genes of yhDFs passed a more stringent threshold of statistical significance (BH‐adjusted *p* value <0.05 and |logFC| >2), further supporting the fact that in old age the adaptation to GDF15 depletion is different from that observed in young individuals (Tables S2–S5). The same holds true for shared genes (Tables 1 and 2). Interestingly, upon GDF15 KD, among the shared upregulated genes we have found *Mmp1, Mmp3*, and *Mmp10* as well as *Pdcd1lg2*. Among the shared downregulated genes, *Sfxn1* and *c‐Kit* were found. These genes are associated with mitochondrial stress and regulation of cell proliferation (Acoba et al., 2021; Foster et al., 2018). A validation analysis by real‐time RT‐PCR of these genes was performed. Among the *Mmp* genes, we selected *Mmp3* as it was the one with the higher log2 fold change. Real‐time RT‐PCR analysis confirmed the results of RNA‐Seq (Figure 5b–i). At basal levels, only the expression level of *Sfxn1* was higher in yhDFs with respect to ohDFs (Figure S2f).

**TABLE 1:** RNA‐Seq list of differentially expressed genes that resulted upregulated in hDFs from both young and old donors.

HGNC_symbol	logFC_Y	*p* value_Y	Adj. *p* value_Y	logFC_O	*p* value_O	Adj. *p* value_O
*P4HA3*	−2.1923	0.0000	0.0017	−1.5491	0.0002	0.2655
*EXTL3*	−1.7178	0.0000	0.0024	−1.1480	0.0002	0.2964
*PLOD2*	−1.9941	0.0000	0.0029	−1.0766	0.0096	0.5603
*PDE4D*	−2.4090	0.0000	0.0030	−1.0674	0.0037	0.4773
*TSPAN13*	−2.1632	0.0000	0.0034	−1.3491	0.0009	0.3263
*ADAMTS6*	−1.3637	0.0000	0.0035	−1.2907	0.0000	0.1531
*COL10A1*	−2.8109	0.0000	0.0046	−1.8200	0.0158	0.6086
*LCP1*	−4.1753	0.0000	0.0050	−2.0088	0.0199	0.6171
*SPP1*	−2.3309	0.0001	0.0073	−2.5595	0.0020	0.4400
*MYEF2*	−1.1623	0.0001	0.0075	−1.1191	0.0042	0.4773
*PTPN22*	−2.4973	0.0001	0.0080	−1.8268	0.0356	0.7021
*MPP4*	−2.7286	0.0002	0.0099	−1.9284	0.0014	0.3834
*MMP3*	−3.2760	0.0003	0.0102	−2.1340	0.0305	0.6816
*PDK3*	−1.4987	0.0003	0.0102	−1.1475	0.0493	0.7718
*GALNT6*	−1.3523	0.0003	0.0107	−1.1686	0.0008	0.3101
*P3H2*	−1.5050	0.0004	0.0116	−1.0127	0.0399	0.7177
*MMP1*	−3.0466	0.0004	0.0126	−2.3632	0.0168	0.6110
*SLC26A4*	−3.3096	0.0007	0.0167	−3.2213	0.0431	0.7336
*HS3ST3A1*	−1.3413	0.0011	0.0201	−1.0392	0.0173	0.6110
*STXBP5‐AS1*	−1.4962	0.0012	0.0216	−1.0677	0.0156	0.6086
*ARG2*	−1.4628	0.0016	0.0245	−1.3881	0.0097	0.5608
*GPR39*	−1.3176	0.0019	0.0269	−1.4884	0.0303	0.6816
*PDCD1LG2*	−1.3894	0.0021	0.0282	−1.0394	0.0283	0.6733
*ARHGAP11B*	−1.5556	0.0035	0.0364	−1.2157	0.0428	0.7334
*LPAR3*	−2.2407	0.0039	0.0391	−2.0386	0.0184	0.6144
*RTL9*	−1.3945	0.0048	0.0440	−1.6841	0.0001	0.2030
*RAB3IP*	−1.1382	0.0055	0.0474	−1.3600	0.0058	0.4904
*MMP10*	−1.5979	0.0058	0.0487	−1.4601	0.0486	0.7659

*Note*: Only the genes with BH‐adjusted *p* value <0.05 in hDFs from young donors are listed in this table.

Abbreviations: Adj. *p* value, BH‐adjusted *p* value; HGNC, gene name according to HUGO Gene Nomenclature Committee; logFC, log2‐transformed fold change; O, in old group; Y, in young group.

**TABLE 2:** RNA‐Seq list of differentially expressed genes that resulted downregulated in hDFs from both young and old donors.

HGNC_symbol	logFC_Y	*p* value_Y	Adj. *p* value_Y	logFC_O	*p* value_O	Adj. *p* value_O
*SFXN1*	1.0844	0.0000	0.0033	1.1452	0.0004	0.3101
*ISY1‐RAB43*	7.9681	0.0000	0.0035	6.8368	0.0412	0.7262
*NYNRIN*	1.1464	0.0000	0.0035	1.0762	0.0005	0.3101
*PPARGC1A*	1.5904	0.0001	0.0068	1.1916	0.0022	0.4400
*KCNS2*	2.0922	0.0001	0.0069	1.6809	0.0009	0.3334
*FGD4*	1.5381	0.0002	0.0102	1.3743	0.0018	0.4207
*PODN*	1.7383	0.0003	0.0102	1.6329	0.0007	0.3101
*KIT*	2.3093	0.0003	0.0115	2.1415	0.0004	0.3101
*TMEM35A*	1.0086	0.0004	0.0118	1.2386	0.0437	0.7361
*MAPT*	1.8248	0.0004	0.0119	1.5075	0.0479	0.7638
*CD34*	1.3339	0.0006	0.0151	2.0415	0.0200	0.6171
*DCN*	1.0076	0.0008	0.0175	1.1757	0.0035	0.4773
*ELFN1*	1.9332	0.0010	0.0197	1.3212	0.0165	0.6110
*TSPAN11*	1.4086	0.0013	0.0220	2.5820	0.0074	0.5296
*PTGIS*	1.0963	0.0015	0.0232	1.0470	0.0001	0.2030
*TAFA5*	1.6712	0.0016	0.0245	1.5207	0.0420	0.7315
*BRINP1*	2.1674	0.0018	0.0261	1.6062	0.0026	0.4530
*FAM43A*	1.0912	0.0020	0.0273	1.0810	0.0131	0.5934
*HMGA2‐AS1*	1.3809	0.0050	0.0449	1.2484	0.0165	0.6110

*Note*: Only the genes with BH‐adjusted *p* value <0.05 in hDFs from young donors are listed in this table.

Abbreviations: Adj. *p* value, BH‐adjusted *p* value; HGNC, gene name according to HUGO Gene Nomenclature Committee; logFC, log2‐transformed fold change; O, in old group; Y, in young group.

## 4 DISCUSSION

In this study we have investigated the role of PLINs in human primary dermal fibroblasts (hDFs) from subjects of different age. First, we have identified PLIN2 and PLIN3 as the sole PLINs that are expressed in this cell type and observed that only PLIN2 is sensitive to lipid overload and responsible for lipid accumulation into LDs. In fact, PLIN2 expression resulted increased upon Oleic Acid (OA) treatment, while PLIN3 expression resulted unchanged upon either PLIN2 downregulation or OA treatment. Interestingly, OA‐induced lipid accumulation resulted impaired when PLIN2 was downregulated. This is in agreement with previous findings indicating that high‐fat diet‐induced steatosis can be prevented by PLIN2 KO (McManaman et al., 2013).

Second, we have observed that the expression level of both PLIN2 and PLIN3 is not modulated by aging in these cells, at least until the age of about 70 years. This is apparently in contrast with our previous data indicating an increase in age of PLIN2 expression in skeletal muscle (SM) and human brain (Conte et al., 2013; Conte, Medici, et al., 2022). It is however to note that all these tissues (hDFs compared to SM and brain) are profoundly different from the point of view of energy metabolism since SM and brain rely much more on lipids than hDFs. It is possible that the age‐related increase in PLIN2 expression observed in SM and human brain is an adaptation to a decreased utilization of lipids for energy production that are thus stored into PLIN2‐coated LDs, or an attempt to avoid free fatty acid toxicity (lipotoxicity).

Third, we could confirm that PLIN2 downregulation is able to impair mitochondrial respiration, as already reported in a different experimental model (pancreatic β cells) (Mishra et al., 2021). Interestingly, the effect is visible only in yhDFs, while ohDFs have a lower basal level of respiration with respect to yhDFs that however is not further decreased upon PLIN2 depletion. This may suggest that the respiration of yhDFs, but not ohDFs, is partly dependent on PLIN2 function. Upon PLIN2 depletion, mitochondrial mass is decreased in both age groups, which could account for the decreased respiratory function, at least for yhDFs. However, the data on ohDFs cast some doubts on this interpretation, as a decrease in mitochondrial mass is present in these cells that is not paralleled by a concomitant decrease in respiration. Therefore, it is possible that other mechanisms take place in determining the observed decrease in respiration. yhDFs and ohDFs apparently implement a different strategy to cope with the consequences of PLIN2 KD, namely an increased turnover (increase in *Fis1* and *Pgc1a* expression) in yhDFs, and an increased fusion (increase in *Opa1* expression) in ohDFs. Further experiments are needed to formally prove this idea, however qualitative data on mitochondrial shape seem to support it.

It is known that mitochondrial dysfunction can elicit retrograde stress responses such as the mitochondrial UPR^mt^ and the integrated stress response (ISR) (Rose et al., 2017). One of the most relevant products of these responses is the mitokine GDF15, a member of the TGF‐β superfamily that includes activins and BMPs. GDF15 is considered a stress responsive cytokine with beneficial effects on health and lifespan in many model organisms (Conte, Giuliani, et al., 2022). Consistently, we have observed an increase in GDF15 expression and secretion upon PLIN2 depletion, and once again this effect was more pronounced in yhDFs, even though ohDFs have much higher basal levels of GDF15 expression. This is in agreement with previous findings that identify GDF15 as one of the most upregulated proteins during aging (Conte, Ostan, et al., 2019). Thus, there is a potential connection between control of lipid metabolism, mitochondria and GDF15, as further suggested by the fact that an elevated expression of GDF15 together with its transcription factors ATF4 and DDIT3 has been found in adipocytes from elderly women characterized by reduced lipogenic capacity and mitochondrial dysfunction (Šrámková et al., 2019). Moreover, GDF15 reduces lipid accumulation thus preventing diseases such as Non‐Alcoholic Fatty Liver Disease (Zhang et al., 2018).

As PLIN2 induces mitochondrial dysfunction that in turn elicits GDF15 production, as mentioned, it seems that GDF15 may be considered as a downstream effector of PLIN2 depletion. In order to investigate this hypothesis, we focused on a well‐known phenomenon linked to mitochondrial dysfunction, that is, cell senescence. In fact, mitochondrial dysfunction is also known to elicit a cell senescence phenomenon (Miwa et al., 2022; Wiley et al., 2016) indicated as MiDAS (mitochondrial dysfunction‐associated senescence) characterized by a peculiar pattern of secretory phenotype (Wiley et al., 2016). As the increase in GDF15 upon PLIN2 KD was more pronounced in yhDFs, we have evaluated whether in these cells there was an induction of cell senescence. In fact, we have observed a higher percentage of β‐Gal‐positive cells and increased expression of *p21* that, however, were not noticed upon PLIN2 + GDF15 KD, suggesting that PLIN2 KD‐induced cell senescence is dependent on GDF15. This leads us to conclude that PLIN2 KD‐mediated cell senescence is dependent on GDF15. It has been reported that GDF15 depletion itself can induce cell senescence, as evaluated by increased expression of SASP components, in particular IL1A, MMP3, MMP10, and MMP12 (Wedel et al., 2023). While we could observe a slight increase in senescent hDFs upon GDF15 KD, this was however not significant and much lower than that induced by PLIN2 KD, we could confirm that an altered gene expression is found upon GDF15 KD, including the upregulation of SASP genes. Considering that the upregulation of metalloproteinases is also found during ER stress (Kim et al., 2010) and that GDF15 KD seems to induce an enlargement of ER compartment, we are tempted to speculate that GDF15 KD could be associated mainly to ER stress rather than cell senescence. This hypothesis is also supported by data showing that Scd‐1 deficiency induces ER stress (Aljohani et al., 2019) and, consistently, we have observed a decrease of *Scd‐1* upon PLIN2 KD, confirming our previous data (Conte, Armani, et al., 2019). Further studies are anyway needed to formally prove this hypothesis.

Finally, we have documented that hDFs from donors of different age do respond differently to PLIN2 KD and GDF15 KD. In particular, ohDFs resulted less affected by PLIN2 KD, but at the same time they have lower basal respiration levels with respect to yhDFs, suggesting that ohDFs are less reliant on PLIN2 than yhDFs for the maintenance of mitochondrial function. The response to GDF15 depletion on gene expression is also profoundly different in the two age groups, with only a minimal part of the affected genes being shared by the two age groups. Moreover, in ohDFs the amplitude of expression changes was much more limited with respect to yhDFs, resulting not significant upon a more stringent analysis. Overall, these findings allow us to speculate that cells from subjects of different age react in a different way to PLIN2 KD, being yhDFs more responsive than ohDFs, even though these latter have a partial mitochondrial impairment that likely is soliciting a chronic stress response with increased production of GDF15. GDF15 in turn has probably more effects than previously thought on cell biology, as it seems to be implicated in determining cell destiny upon mitochondrial stress. However, once again, in cells from old subjects that are under chronic stress, GDF15 seems unable to elicit the same responses as in cells from young subjects. The reasons for this age‐dependent difference in GDF15 activity are unclear and further studies are needed to clarify this aspect, including gene expression studies upon GDF15 upregulation.

In conclusion, the present data confirm the emerging role of LDs for cell biology where the LD‐coating proteins like PLINs play a so far underestimated role. In particular, PLIN2 is emerging as a key player, as demonstrated in different experimental models (Conte et al., 2013; Conte, Medici, et al., 2022; McManaman et al., 2013). Our data add further knowledge, by indicating that PLIN2 appears as the predominant PLIN in hDFs and its presence is needed to save cells from mitochondrial impairment. It is not yet clear whether mitochondrial dysfunction is directly caused by a PLIN2 KD‐mediated missed contact with LDs. As it seems that PLIN2 KD causes a decrease in LD content, it is also possible that the effect on mitochondria is indirect, as it has been reported that LDs have a cytoprotective role as they can serve as “sink” not only for toxic lipids but also for damaged/misfolded/aggregated mitochondrial proteins, at least in yeast (Geltinger et al., 2020). Interestingly, these findings resulted more pronounced in yhDFs, while in ohDFs there was only a trend even though these latter have a higher basal level of mitochondrial dysfunction and GDF15 expression. It is possible that this is due to a phenomenon of adaptation by which cells from old subjects become less responsive or even resistant to stress signaling, like GDF15. To this regard, GDF15 emerges as a downstream mediator of PLIN2 KD‐induced effects like cell senescence. This is a potentially far‐reaching connection, as it may represent a link between LD biology and stress responses. There is in fact a great interest on the role of both LDs and GDF15 in human aging and age‐related diseases, as well as their possible pharmacological targeting (Conte, Giuliani, et al., 2022). Further studies are needed to better clarify this intricate connection.

## AUTHOR CONTRIBUTIONS

AC and LR performed and conceived experiments, performed statistical analyses, and wrote the manuscript. SV and GP performed transmission electron microscopy analysis and revised the manuscript. MS, LI, and AMP performed measurement and analysis of mitochondrial respiration and revised the manuscript. MT and GC performed TBARS analysis and statistical analysis. KK and PG performed RNA‐Seq data analysis. AS critically discussed the results. SS and MC carried out the design of the study, critically analyzed the results, and wrote the manuscript. All authors contributed to the article and approved the submitted version.

## FUNDING INFORMATION

The work has been co‐funded from Next Generation EU, in the context of the National Recovery and Resilience Plan, Investment PE8 – Project Age‐It: “Ageing Well in an Ageing Society” to SS. This resource was co‐financed by the Next Generation EU [DM 1557 11.10.2022]. The work has been also co‐funded from CN3 – Gene Therapy and Drug Development with RNA Technology: “Preclinical testing of nuclease and RNA drugs for quantitative and qualitative mtDNA defects” to LI. The views and opinions expressed are only those of the authors and do not necessarily reflect those of the European Union or the European Commission. Neither the European Union nor the European Commission can be held responsible for them. The work was also co‐funded from the Italian Ministry of University and Research (MUR) PRIN 2022, Project # 2022KS8T4N “GDF15 as a key player and a potential target to tackle ageing and age‐associated diseases: an in silico, in vitro and ex vivo study” to SS, JPI‐HDHL‐Metadis, “EURODIET” project (ID: 1164; 2020–2023) to AS and “PRIN2020 – Italian Ministry of University and Research – 2020RRJP5L_004” to AMP.

## CONFLICT OF INTEREST STATEMENT

All authors declared no financial or scientific conflicts of interest with regard to the research described in this manuscript.

## CONSENT TO PARTICIPATE

Signed informed consent was obtained from all individual participants who donated cells included in the study.

## Supporting information


Figure S1.

Figure S2.

Figure S3.

Figure S4.

Figure S5.

Figure S6.

Figure S7.

Table S1.

Figure Captions.

## Data Availability

Datasets generated during and/or analyzed during the current study are not publicly available but are available from the corresponding author on reasonable request.
