# Supplementary figures and images for: Downregulation of PLIN2 in human dermal fibroblasts impairs mitochondrial function in an age‐dependent fashion and induces cell senescence via GDF15
Source: Aging Cell. 2024 Apr 22;23(5):e14111. doi: 10.1111/acel.14111 (PMC11113257; doi:10.1111/acel.14111)

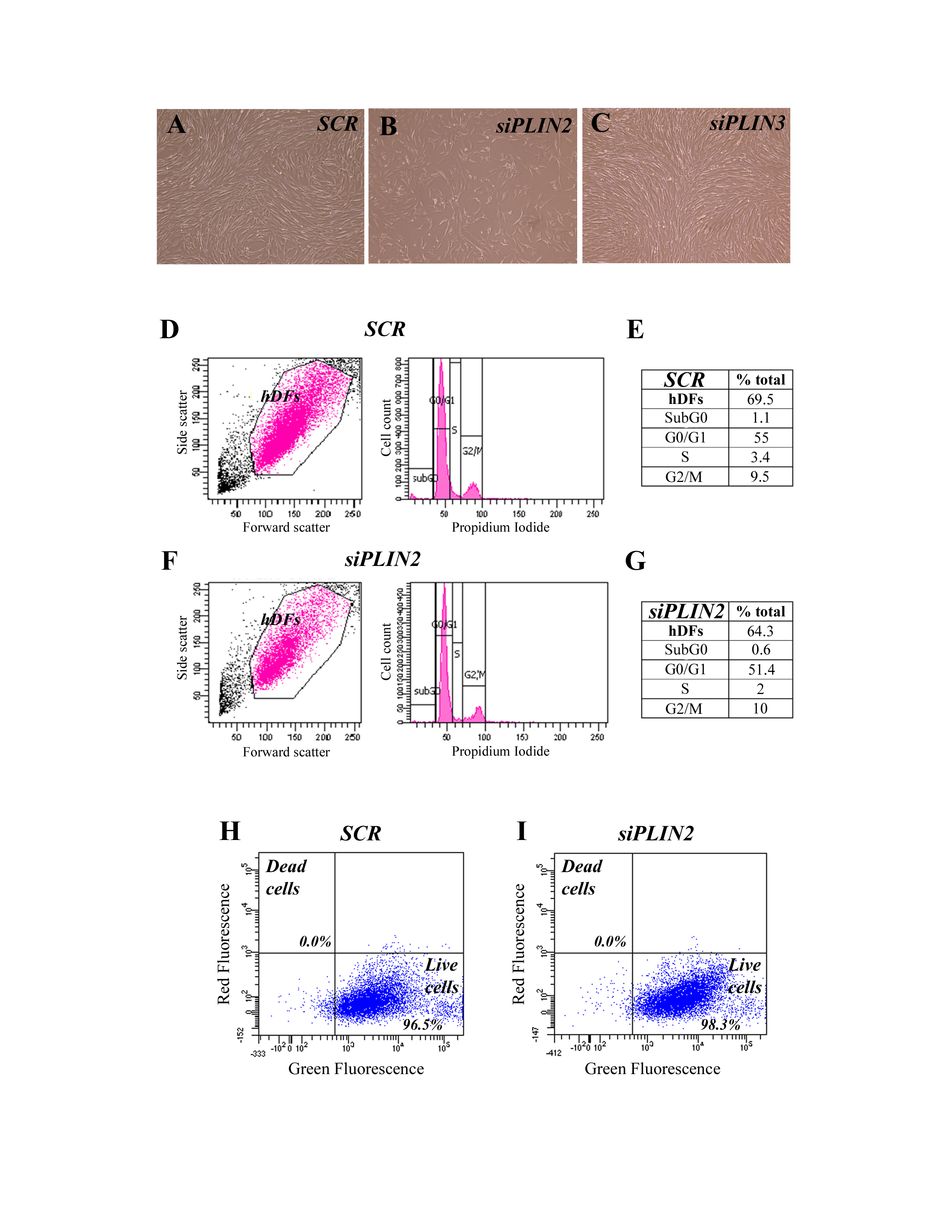

Supplement: Supplementary file 1 — Figure S1. [file ACEL-23-e14111-s001.tif]

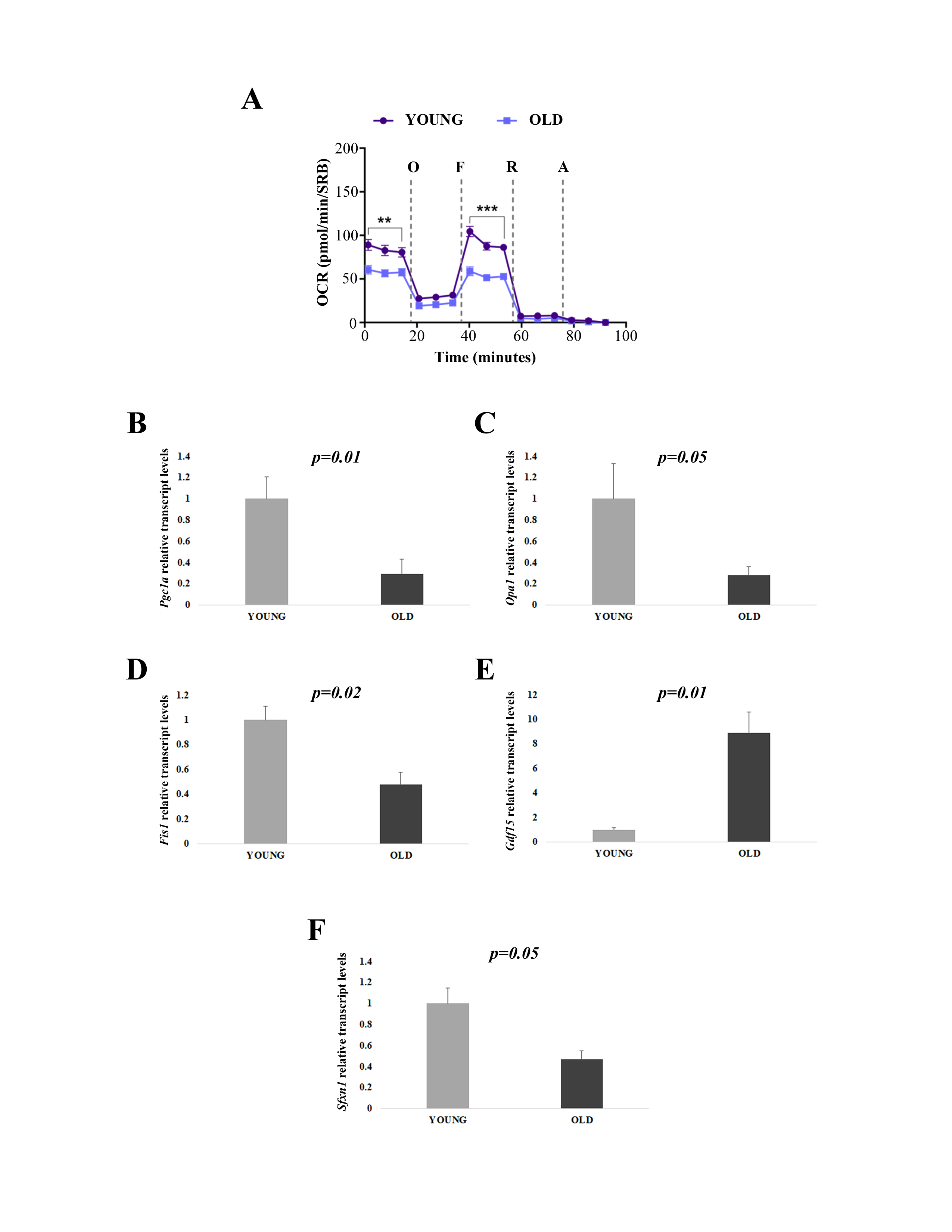

Supplement: Supplementary file 2 — Figure S2. [file ACEL-23-e14111-s009.tif]

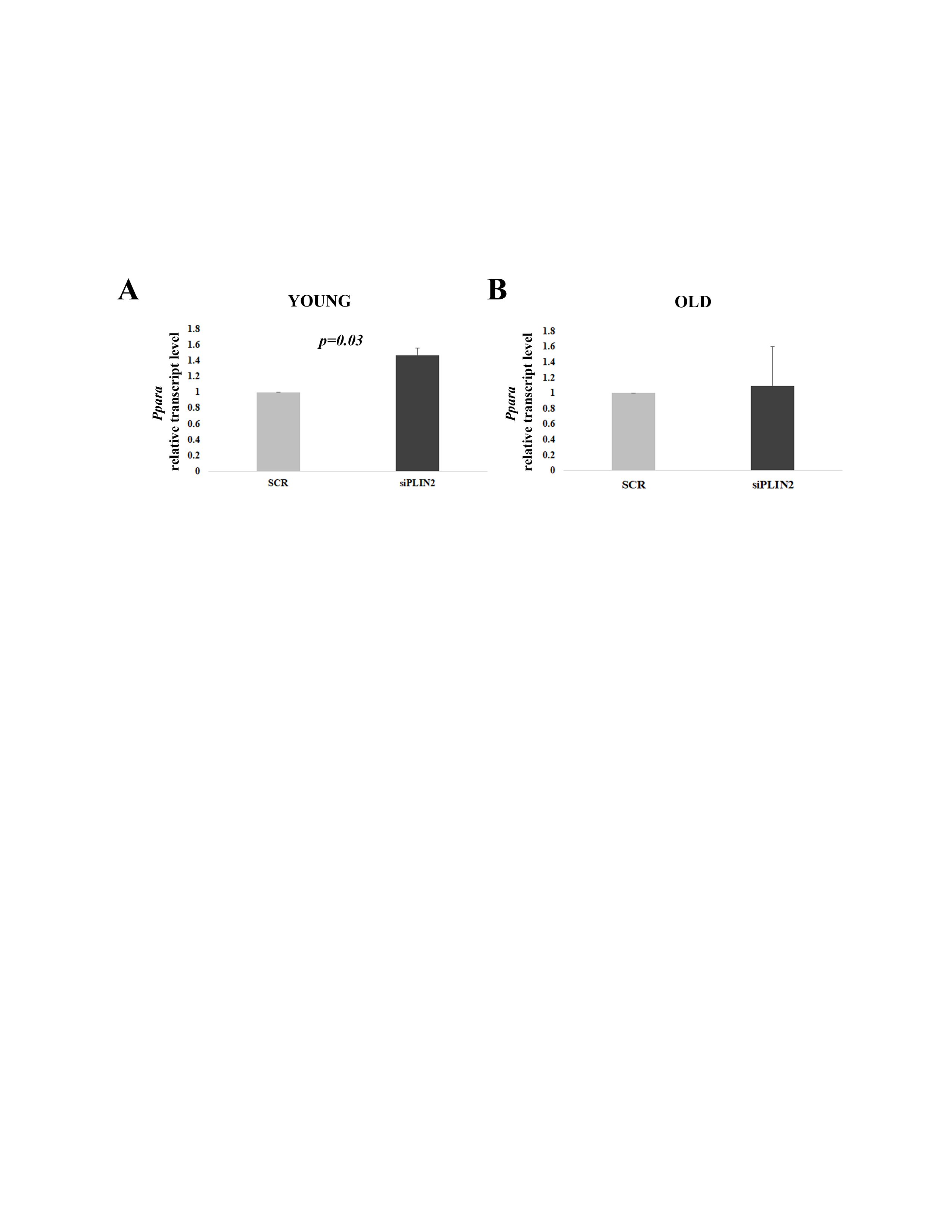

Supplement: Supplementary file 3 — Figure S3. [file ACEL-23-e14111-s006.tif]

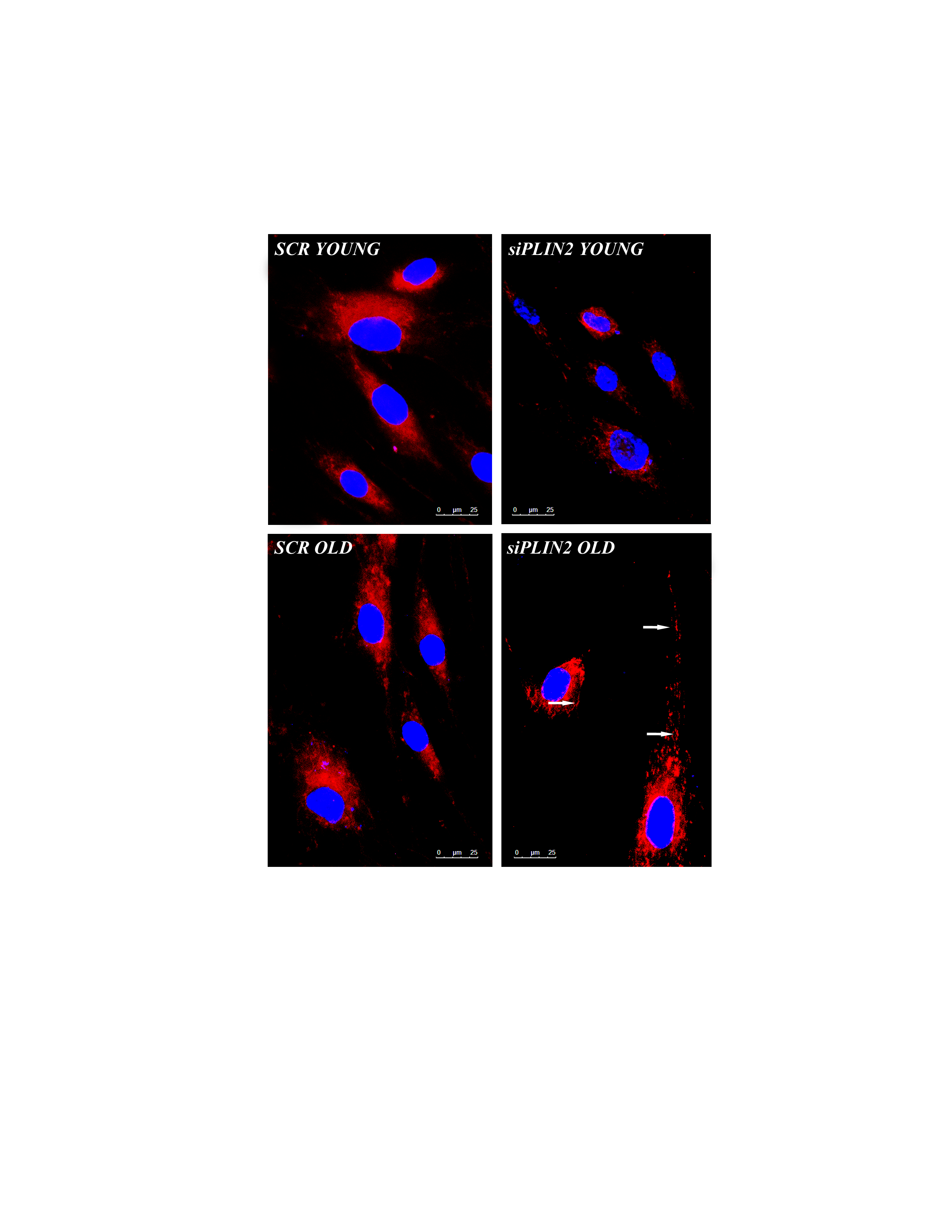

Supplement: Supplementary file 4 — Figure S4. [file ACEL-23-e14111-s004.tiff]

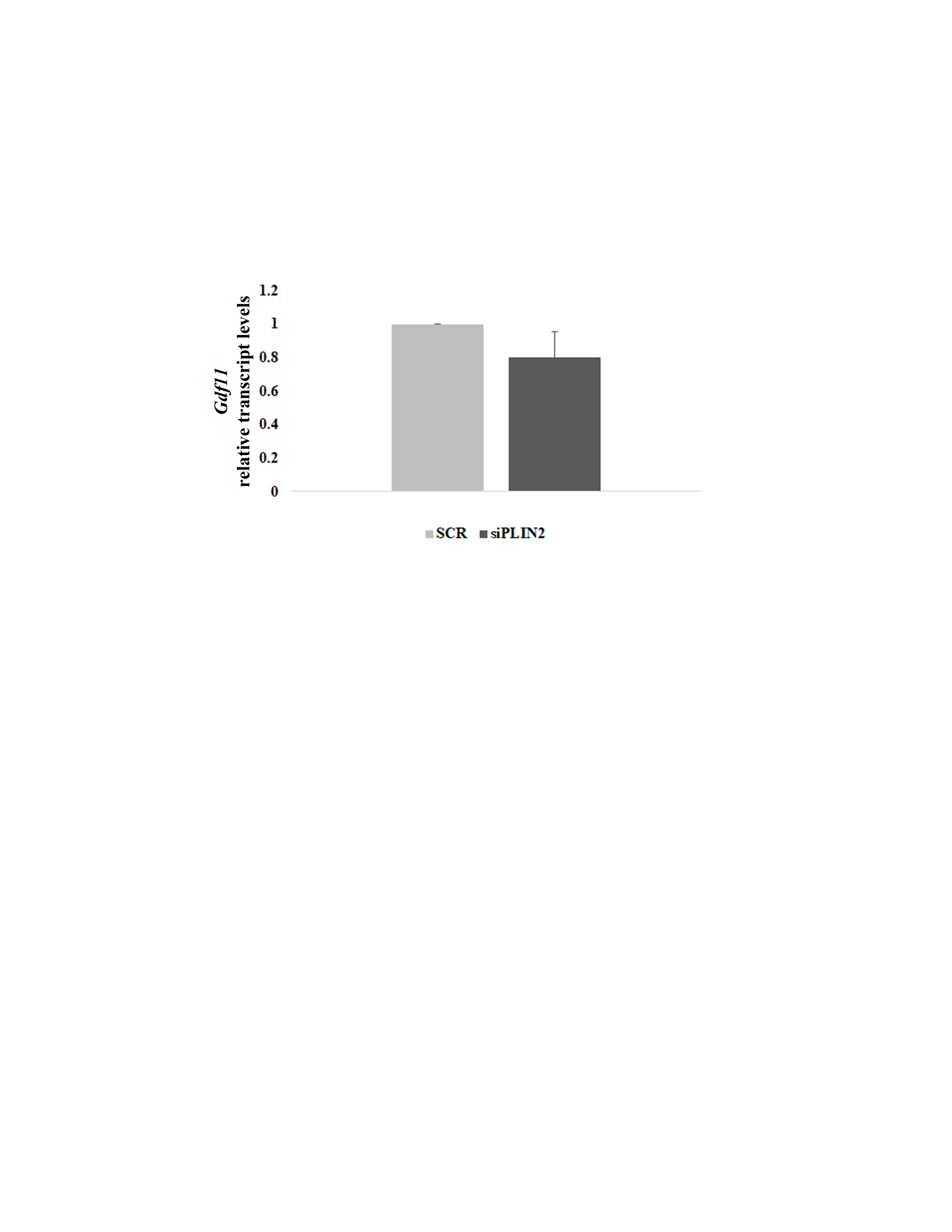

Supplement: Supplementary file 5 — Figure S5. [file ACEL-23-e14111-s007.tiff]

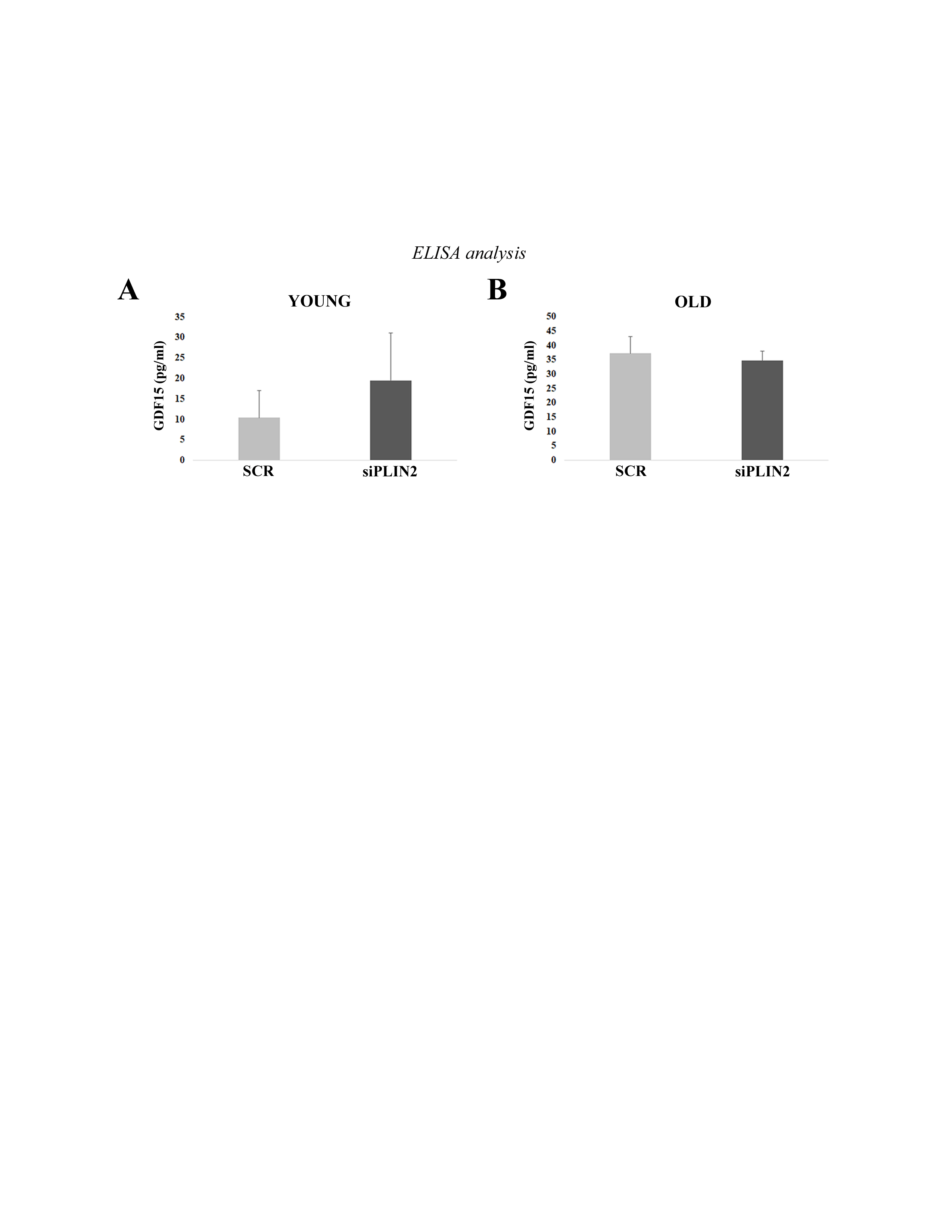

Supplement: Supplementary file 6 — Figure S6. [file ACEL-23-e14111-s002.tiff]

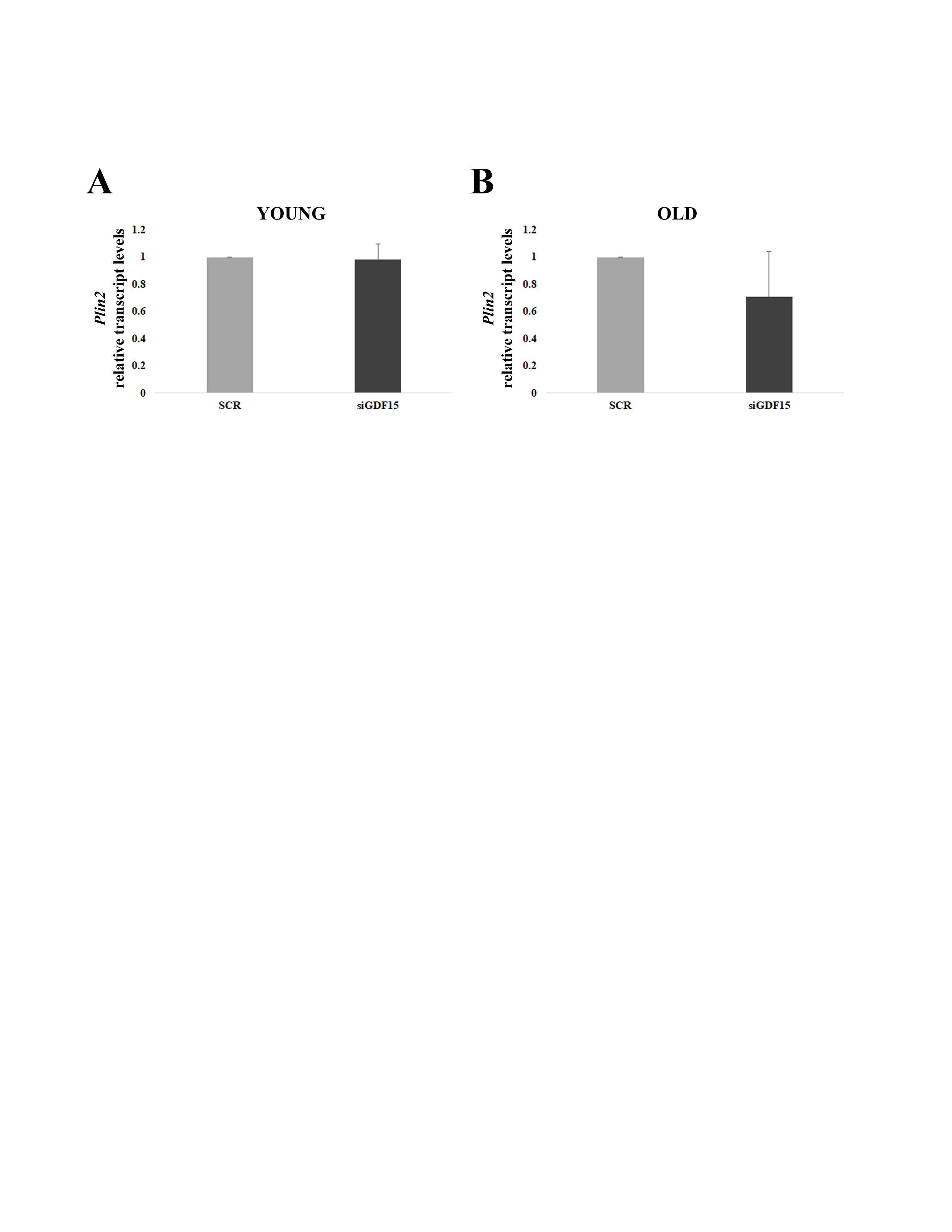

Supplement: Supplementary file 7 — Figure S7. [file ACEL-23-e14111-s003.tiff]
