# Supplementary material for: Downregulation of PLIN2 in human dermal fibroblasts impairs mitochondrial function in an age‐dependent fashion and induces cell senescence via GDF15
Source: Aging Cell. 2024 Apr 22;23(5):e14111. doi: 10.1111/acel.14111 (PMC11113257; doi:10.1111/acel.14111)
Supplement: Supplementary file 8 — Table S1. [file ACEL-23-e14111-s005.docx]

**SUPPLEMENTARY TABLES**

**Supplementary Table 1**

List of primary antibodies used

| **Primary antibody** | **Catalog number** | **Supplier** | **WB dilution** |
| --- | --- | --- | --- |
| GAPDH | NB300-221 | Novus Biological | 1:20,000 |
| PLIN1 | 9349 | Cell Signaling Technology | 1:1000 |
| PLIN2 | LS-B4850 | LifeSpan BioSciences, Inc. | 1:1000 |
| PLIN3 | LS-B2539 | LifeSpan BioSciences, Inc. | 1:1000 |
| PLIN4 | NBP2-13776 | Novus Biological | 1:1000 |
| PLIN5 | NB110-60509 | Novus Biological | 1:1000 |
| VDAC1 | ab14734 | Abcam | 1:2500 |

**Supplementary Table 2**

RNA-seq list of differentially expressed genes that resulted upregulated exclusively in hDFs from young donors. Only the genes with BH-adjusted p-value<0.05 in hDFs from young donors are listed in this table (HGNC: gene name according to HUGO Gene Nomenclature Committee; logFC: Log2-transformed fold change; Adj.P-Value: BH-adjusted p-value)

| **HGNC _symbol** | **logFC** | **P-Value** | **Adj.P-Value** | |
| --- | --- | --- | --- | --- |
| PPAN-P2RY11 | -7.0093 | 9.21E-06 | 0.0035 |  |
| AFP | -3.72315 | 9.02E-05 | 0.0072 |  |
| DNER | -2.22773 | 0.000212 | 0.0098 |  |
| DELEC1 | -4.54639 | 0.000236 | 0.0101 |  |
| TMEM71 | -2.39479 | 0.000283 | 0.0104 |  |
| TACR1 | -4.2971 | 0.00036 | 0.0117 |  |
| ANAPC1P2 | -4.0943 | 0.000378 | 0.0119 |  |
| CAPSL | -4.38867 | 0.000386 | 0.0121 |  |
| CDRT1 | -2.50263 | 0.000414 | 0.0124 |  |
| C10orf88B | -3.27267 | 0.000472 | 0.0132 |  |
| CA9 | -3.86006 | 0.000488 | 0.0134 |  |
| MYOSLID | -2.22819 | 0.000616 | 0.0151 |  |
| MYO5C | -3.80376 | 0.000663 | 0.0158 |  |
| NMRAL2P | -2.48327 | 0.000739 | 0.0166 |  |
| C2CD4D-AS1 | -3.28303 | 0.00108 | 0.0202 |  |
| PPP1R36 | -2.79045 | 0.001113 | 0.0205 |  |
| TRIM36 | -2.21394 | 0.001424 | 0.0229 |  |
| TSPAN12 | -2.52788 | 0.001563 | 0.0236 |  |
| BEST3 | -3.69 | 0.001649 | 0.0245 |  |
| RAPGEF4 | -3.4643 | 0.002887 | 0.033 |  |
| PANX2 | -2.51967 | 0.003142 | 0.0346 |  |
| COLEC10 | -2.77618 | 0.00331 | 0.0356 |  |
| CALHM6 | -3.75684 | 0.003428 | 0.0361 |  |
| TREML3P | -3.36913 | 0.003436 | 0.0361 |  |
| LINC02015 | -2.15912 | 0.003833 | 0.0383 |  |
| ACP3 | -2.61665 | 0.004077 | 0.0399 |  |
| KIAA0319 | -3.75052 | 0.004431 | 0.0418 |  |
| FPR1 | -3.02432 | 0.004622 | 0.0428 |  |
| GKAP1 | -2.33858 | 0.005015 | 0.0449 |  |
| SERPINB4 | -3.35729 | 0.005187 | 0.046 |  |
| AS3MT | -3.19488 | 0.005369 | 0.0469 |  |
| LINC00707 | -2.37892 | 0.00538 | 0.0469 |  |
| CENPM | -2.12681 | 0.005832 | 0.0487 |  |
| MRPS31P5 | -2.6513 | 0.006027 | 0.0497 |  |

**Supplementary Table 3**

RNA-seq list of differentially expressed genes that resulted downregulated exclusively in hDFs from young donors. Only the genes with BH-adjusted p-value<0.05 in hDFs from young donors are listed in this table (HGNC: gene name according to HUGO Gene Nomenclature Committee; logFC: Log2-transformed fold change; Adj.P-Value: BH-adjusted p-value)

| **HGNC _symbol** | **logFC** | **P-Value** | **Adj.P-Value** |
| --- | --- | --- | --- |
| OSR2 | 2.07800 | 6.45E-07 | 0.00168 |
| VWCE | 2.46378 | 1.73E-05 | 0.00433 |
| GDF15 | 2.70175 | 0.000106294 | 0.00748 |
| BST2 | 2.18716 | 0.000104816 | 0.00748 |
| TMEM255A | 5.27367 | 0.000177334 | 0.00906 |
| ADAMDEC1 | 4.37285 | 0.000183424 | 0.00918 |
| CD36 | 2.24959 | 0.000559637 | 0.01441 |
| PIK3R2 | 6.12979 | 0.00064699 | 0.01549 |
| NTRK2 | 3.82572 | 0.00157491 | 0.02378 |
| RPS26P19 | 3.01264 | 0.001745809 | 0.02533 |
| BORCS7-ASMT | 6.90660 | 0.001766343 | 0.02546 |
| VWA1 | 3.70782 | 0.002414403 | 0.03007 |
| PRSS27 | 2.22375 | 0.003166678 | 0.03473 |
| KLHL30 | 3.86918 | 0.003731257 | 0.03778 |
| ACAN | 2.84837 | 0.004123884 | 0.04016 |
| FXYD1 | 2.33702 | 0.005305665 | 0.04668 |
| EPHB6 | 2.03079 | 0.00547578 | 0.04739 |

**Supplementary Table 4**

RNA-seq list of differentially expressed genes that resulted upregulated exclusively in hDFs from old donors. Only the genes with |logFC| > 2 in hDFs from old donors are listed in this table (HGNC: gene name according to HUGO Gene Nomenclature Committee; logFC: Log2-transformed fold change; Adj.P-Value: BH-adjusted p-value)

| **HGNC _symbol** | **logFC** | **P-Value** | **Adj.P-Value** |
| --- | --- | --- | --- |
| FMC1-LUC7L2 | -7.489 | 0.00273 | 0.45307147 |
| TVP23C-CDRT4 | -6.4098 | 0.00384 | 0.47725286 |
| DNAJC12 | -3.9831 | 0.00034 | 0.31014782 |
| ARPIN-AP3S2 | -3.8957 | 0.04819 | 0.7656691 |
| ATP2A1-AS1 | -3.6681 | 0.00527 | 0.48118436 |
| AMY2A | -3.5718 | 0.00661 | 0.51042567 |
| MRPS24 | -3.5266 | 0.02965 | 0.68054758 |
| UCN2 | -3.1298 | 0.00699 | 0.5170953 |
| LINC02085 | -2.9298 | 0.00894 | 0.55005694 |
| NEDD8-MDP1 | -2.8143 | 0.04723 | 0.76095465 |
| VWF | -2.7888 | 0.04954 | 0.7731232 |
| CCDC81 | -2.7146 | 0.04799 | 0.76403058 |
| CXADR | -2.6031 | 0.03866 | 0.71232518 |
| ZNF670-ZNF695 | -2.5951 | 0.04363 | 0.73608322 |
| TICAM2 | -2.5745 | 0.03415 | 0.69654752 |
| CFAP45 | -2.5434 | 0.00224 | 0.43998518 |
| RSAD2 | -2.1081 | 0.00119 | 0.36350464 |
| FBXW10 | -2.0892 | 0.01061 | 0.58249537 |
| CXCL11 | -2.0684 | 0.01383 | 0.59344143 |
| CITED4 | -2.0157 | 0.0416 | 0.7264069 |
| OASL | -2.0104 | 0.038 | 0.71029297 |

**Supplementary Table 5**

RNA-seq list of differentially expressed genes that resulted downregulated exclusively in hDFs from old donors. Only the genes with |logFC| > 2 in hDFs from old donors are listed in this table (HGNC: gene name according to HUGO Gene Nomenclature Committee; logFC: Log2-transformed fold change; Adj.P-Value: BH-adjusted p-value)

| **HGNC _symbol** | **logFC** | **P-Value** | **Adj.P-Value** |
| --- | --- | --- | --- |
| HNRNPUL2-BSCL2 | 7.40756 | 0.0021 | 0.43998518 |
| C1QTNF3-AMACR | 6.35182 | 0.00879 | 0.55005694 |
| ENTPD3 | 4.98277 | 0.00022 | 0.29635867 |
| ATAD3C | 4.89066 | 0.00455 | 0.47725286 |
| MROH7-TTC4 | 4.78313 | 4.5E-05 | 0.20020527 |
| ACSL5 | 4.54866 | 0.00096 | 0.3357937 |
| ZNF564 | 4.5167 | 0.01726 | 0.61102535 |
| CELF6 | 4.42127 | 0.01956 | 0.61548324 |
| ADCY5 | 4.2558 | 0.0013 | 0.37074234 |
| CARD9 | 4.24705 | 0.01732 | 0.61102535 |
| CHL1 | 3.67198 | 0.00406 | 0.47725286 |
| SHC2 | 3.66059 | 0.00331 | 0.47620974 |
| MSL3P1 | 3.63832 | 0.01357 | 0.59344143 |
| IGF1 | 3.41926 | 0.00878 | 0.55005694 |
| CCDC17 | 3.40806 | 0.00535 | 0.48157743 |
| PTH1R | 3.33586 | 0.00636 | 0.50331082 |
| ADH7 | 3.30457 | 0.00458 | 0.47725286 |
| MPP7 | 2.97654 | 0.02189 | 0.63140588 |
| SLC16A14 | 2.91509 | 0.02217 | 0.63140588 |
| CENPS-CORT | 2.90705 | 0.01387 | 0.59344143 |
| TMEM176A | 2.81844 | 0.00812 | 0.54563856 |
| NXNL2 | 2.78159 | 0.01428 | 0.59642001 |
| TRIM59 | 2.77843 | 0.04034 | 0.72223198 |
| PRKCZ | 2.73832 | 0.00421 | 0.47725286 |
| LINC00840 | 2.73209 | 0.0043 | 0.47725286 |
| LINC01285 | 2.72518 | 0.01845 | 0.61439633 |
| PDE1A | 2.70639 | 0.00891 | 0.55005694 |
| LINC01260 | 2.70421 | 0.04453 | 0.73962539 |
| PMEL | 2.66075 | 0.00396 | 0.47725286 |
| TACR3 | 2.64934 | 0.04101 | 0.72624437 |
| NPIPB1P | 2.60242 | 0.02332 | 0.64384772 |
| TMEM176B | 2.50932 | 0.02225 | 0.63140588 |
| CATSPER1 | 2.44679 | 0.01377 | 0.59344143 |
| SFRP4 | 2.44182 | 0.01733 | 0.61102535 |
| ITGB8-AS1 | 2.3431 | 0.03416 | 0.69654752 |
| CFAP206 | 2.13724 | 0.02438 | 0.65327672 |
| HTR2B | 2.09968 | 0.01397 | 0.59344143 |
| LINC01391 | 2.09953 | 0.04083 | 0.72624437 |
| EGFL8 | 2.09377 | 0.04751 | 0.76191015 |
