# Supplementary material for: Downregulation of PLIN2 in human dermal fibroblasts impairs mitochondrial function in an age‐dependent fashion and induces cell senescence via GDF15
Source: Aging Cell. 2024 Apr 22;23(5):e14111. doi: 10.1111/acel.14111 (PMC11113257; doi:10.1111/acel.14111)
Supplement: Supplementary file 9 — Figure Captions. [file ACEL-23-e14111-s008.docx]

**Suppl. Figure 1** Light microscopy and flow cytometry analyses of hDFs after RNA interference experiments. (A-C) Representative light microscopy images of hDFs treated with (A) scramble (SCR), (B) siPLIN2 and (C) siPLIN3. (D-G) Flow cytometry analysis of cell cycle by propidium iodide staining. Data obtained from a representative yhDF sample out of three, transfected with scramble (SCR) and siPLIN2, are shown. Electronic gating on FSC and SSC parameters (magenta region) was used to exclude cell debris. (H-I) Flow cytometry viability analysis using LIVE/DEAD viability/cytotoxicity kit. Data obtained from a representative yhDF sample out of three are shown.

**Suppl. Figure 2** Oxygen consumption rate (OCR) profile and gene expression in hDFs from 6 young and 5 old donors. (A) OCR profile. (B-F) Real Time RT-PCR analysis of *Pgc1α*, *Opa1*, *Fis1* and *Sfxn1* expression. Data are expressed as mean ± SE. Student’s t test was applied.

**Suppl. Figure 3** Real Time RT-PCR analysis of *Ppara* expression after PLIN2 KD in hDFs from (A) 3 young and (B) 3 old donors. Data are expressed as mean ± SE. Student’s t test was applied

**Suppl. Figure 4** MitoTracker Red staining of hDFs from one young and one old donor. Upon PLIN2 downregulation, yhDFs display punctated mitochondria, while ohDFs display some elongated mitochondria (arrows). Representative images out of 6 to 10 fields for each condition are shown.

**Suppl. Figure 5** Real Time RT-PCR analysis of *Gdf11* expression in 11 hDFs samples, from young and old donors, after PLIN2 downregulation (siPLIN2) compared to scramble-treated samples (SCR). Data are expressed as mean ± SE. Student’s t test was applied

**Suppl. Figure 6** GDF15 analysis after PLIN2 downregulation. ELISA analysis of GDF15 secretion in the culture media of hDFs from (A) 6 young and (B) 5 old donors, expressed as absolute concentration (pg/mL). Data are expressed as mean ± SE. Mann-Whitney U Test was applied

**Suppl. Figure 7** Real Time RT-PCR analysis of *Plin2* expression after GDF15 KD in hDFs from (A) 3 young and (B) 3 old donors. Data are expressed as mean ± SE. Student’s t test was applied
